# Systematic characterization of seed overlap microRNA cotargeting associated with lupus pathogenesis

**DOI:** 10.1186/s12915-022-01447-4

**Published:** 2022-11-11

**Authors:** Hiroki Kitai, Noritoshi Kato, Koichi Ogami, Shintaro Komatsu, Yu Watanabe, Seiko Yoshino, Eri Koshi, Shoma Tsubota, Yoshio Funahashi, Takahiro Maeda, Kazuhiro Furuhashi, Takuji Ishimoto, Tomoki Kosugi, Shoichi Maruyama, Kenji Kadomatsu, Hiroshi I. Suzuki

**Affiliations:** 1 Department of Nephrology, Nagoya University Graduate School of Medicine, 65 Tsurumai-cho, Showa-ku, Nagoya, Aichi 466-8550 Japan; 2 Division of Molecular Oncology, Center for Neurological Diseases and Cancer, Nagoya University Graduate School of Medicine, 65 Tsurumai-cho, Showa-ku, Nagoya, Aichi 466-8550 Japan; 3 Department of Biochemistry, Nagoya University Graduate School of Medicine, 65 Tsurumai-cho, Showa-ku, Nagoya, Aichi 466-8550 Japan; 4 Present Address: Yoshio Funahashi, Department of Anesthesiology and Perioperative Medicine, Oregon Health and Science University, 3181 S.W. Sam Jackson Park Road, Portland, OR 97239 USA; 5 Department of General Medicine, Nagasaki University Graduate School of Biomedical Sciences, 1-7-1 Sakamoto, Nagasaki, Nagasaki 852-8501 Japan; 6 Present Address: Takuji Ishimoto, Department of Nephrology and Rheumatology, Aichi Medical University, 1-1 Yazakokarimata, Nagakute, Aichi 480-1195 Japan; 7 Institute for Glyco-core Research (iGCORE), Nagoya University, Furo-cho, Chikusa-ku, Nagoya, Aichi 464-8601 Japan

**Keywords:** Cotargeting, Evolution, MicroRNA, Seed, SLE

## Abstract

**Background:**

Combinatorial gene regulation by multiple microRNAs (miRNAs) is widespread and closely spaced target sites often act cooperatively to achieve stronger repression (“neighborhood” miRNA cotargeting). While miRNA cotarget sites are suggested to be more conserved and implicated in developmental control, the pathological significance of miRNA cotargeting remains elusive.

**Results:**

Here, we report the pathogenic impacts of combinatorial miRNA regulation on inflammation in systemic lupus erythematosus (SLE). In the SLE mouse model, we identified the downregulation of two miRNAs, miR-128 and miR-148a, by TLR7 stimulation in plasmacytoid dendritic cells. Functional analyses using human cell lines demonstrated that miR-128 and miR-148a additively target KLF4 via extensively overlapping target sites (“seed overlap” miRNA cotargeting) and suppress the inflammatory responses. At the transcriptome level, “seed overlap” miRNA cotargeting increases susceptibility to downregulation by two miRNAs, consistent with additive but not cooperative recruitment of two miRNAs. Systematic characterization further revealed that extensive “seed overlap” is a prevalent feature among broadly conserved miRNAs. Highly conserved target sites of broadly conserved miRNAs are largely divided into two classes—those conserved among eutherian mammals and from human to *Coelacanth*, and the latter, including KLF4-cotargeting sites, has a stronger association with both “seed overlap” and “neighborhood” miRNA cotargeting. Furthermore, a deeply conserved miRNA target class has a higher probability of haplo-insufficient genes.

**Conclusions:**

Our study collectively suggests the complexity of distinct modes of miRNA cotargeting and the importance of their perturbations in human diseases.

**Supplementary Information:**

The online version contains supplementary material available at 10.1186/s12915-022-01447-4.

## Background

MicroRNAs (miRNAs) are small non-coding RNAs of approximately 22 nucleotides (nt) in length that bind to the 3′ untranslated regions (3′ UTRs) of target mRNAs and repress them [1]. Target recognition by miRNAs is mediated by the interaction between the seed sequences of miRNAs (nucleotides 2–8) and the complementary sequences within 3′ UTRs. Typically, each conserved miRNA has hundreds of conserved miRNA target sites but mediates only modest target repression via single sites. To establish robust repression, multiple target sites and miRNAs are proposed to act cooperatively [2]. Although repression by multiple distinct sites is usually log-additive, closely spaced miRNA sites with a distance of ~ 15–100 nt between seed starts often act synergistically (“neighborhood” miRNA cotargeting) [3–6]. Consistent with this, as for conserved miRNAs, conserved and closely spaced sites for identical miRNAs are overrepresented than expected by chance, whereas the absolute number of such sites is low [4, 5]. This suggests selective maintenance of “neighborhood” miRNA cotargeting. Closely connected genes are also frequently cotargeted by multiple distinct miRNAs [7]. In addition, a recent study showed that hundreds of pairs of distinct miRNAs, especially pairs of brain-enriched miRNAs, share more mRNA targets than expected by chance [8]. These findings highlight the importance of miRNA cotargeting in conserved miRNA function and development.

A series of animal studies demonstrated the biological importance of miRNAs. In mice, knockout of many conserved miRNAs results in abnormal knockout phenotypes, including various developmental defects and disease conditions including immune disorders [1]. Tissue-specific miRNAs, which show such abnormal knockout phenotypes upon depletion, are frequently associated with tissue-specific super-enhancers [9]. By contrast, and reflecting the complex interplay and redundancy among multiple miRNA family members, the phenotypic changes are often context-dependent and become apparent upon the disruption of multiple family members. For example, combinatorial deletion of the miR-17~92 polycistronic cluster and related miRNA clusters [10], and targeted deletion of individual miRNAs within the miR-17~92 cluster [11], revealed functional cooperation among multiple miRNA clusters, and among individual miRNAs, as well as context-dependent roles of individual miRNAs. On the other hand, the crosstalk between multiple miRNAs and target sites and the underlying mechanism(s) remain elusive in various pathological conditions, although dysregulation of multiple miRNAs is frequent in various diseases.

miRNAs have important roles in various inflammatory diseases, including autoimmune diseases. Systemic lupus erythematosus (SLE) is an autoimmune disease, in which the deposition of immune complexes (IC) occurs in multiple organs due to the production of autoantibodies [12]. In particular, about 40–70% of patients develop lupus nephritis due to IC deposition in the kidney, and 10–30% of these patients progress to end-stage renal disease despite immunosuppressive treatment. Plasmacytoid dendritic cells (pDCs) are key regulators of innate immunity and produce type I interferon (IFN) and other inflammatory cytokines, including IL-6 and TNF-α, following recognition of viral nucleic acids by intracellular Toll-like receptor 7/9 (TLR7/9) [13]. Recently, it has been shown that pDCs have critical roles in SLE by secreting type I IFN, IL-6, and TNF-α upon stimulation with IC composed of autoantibodies and self-nucleic acid [14–16]. Indeed, ablation of pDCs in SLE model mice improved SLE-associated lupus nephritis [17–19]. Although the alteration of multiple miRNAs in various immune cells has been implicated in SLE [20], alterations of miRNAs in pDCs have not been explored in depth, with a few exceptions [21–23].

In this study, we performed miRNA expression profiling of the SLE mice model and identified the downregulation of two miRNAs, miR-128 and miR-148a, in pDCs. Mechanistically, miR-128 and miR-148a target Kruppel-like factor 4 (KLF4) via extensively overlapped target sites and negatively regulate inflammatory cytokine production. In RNA-seq analysis, such “seed overlap” cotarget sites show increased susceptibility to downregulation over non-overlapping sites upon overexpression of two miRNAs. This suggests that “seed overlap” miRNA cotargeting increases susceptibility to multiple miRNAs, while “neighborhood” miRNA cotargeting cooperatively enhances target repression. We further expanded these findings by integratively analyzing the seed overlap patterns of all miRNAs and conservation patterns of “seed overlap” target sites. The integrative bioinformatics analysis also uncovered two major classes of highly conserved sites of broadly conserved miRNAs in mammals and their unique relationships with RNA repression, miRNA cotargeting, and haplo-insufficiency of target genes. These findings provide unique insight into the complicated aspects of cotargeting and conservation of miRNA target sites.

## Results

### Downregulation of miR-128 and miR-148a in splenic pDCs of the IMQ-induced SLE mouse model

**Fig. 1:**
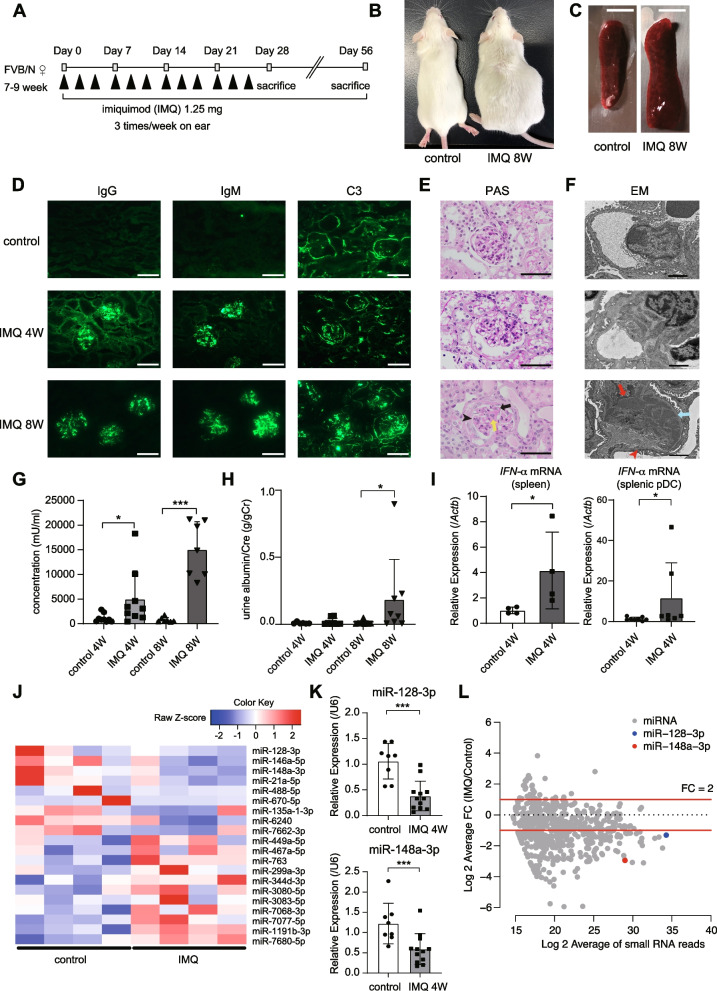
IMQ-induced SLE mouse model: downregulation of miR-128-3p and miR-148a-3p in splenic pDCs. **A** Experimental protocol of the IMQ model. **B**, **C** Massive ascites (**B**) and splenomegaly (**C**) in IMQ-treated mice at 8 weeks. Bar = 0.5 cm. **D** Immunofluorescence image of a kidney section showing IgG, IgM, and C3 in a 4-week-old control mouse (upper), 4-week-old IMQ mouse (middle), and 8-week-old IMQ mouse (lower). Bar = 100 μm. **E** Periodic acid-Schiff (PAS) staining of a kidney section of a 4-week-old control mouse (upper), 4-week-old IMQ mouse (middle), and 8-week-old IMQ mouse (lower). Bar = 100 μm. **F** Electron microscope image of the kidney section of a 4-week-old control mouse (upper), 4-week-old IMQ mouse (middle), and 8-week-old IMQ mouse (lower). Bar = 2.0 μm. **G** Anti-ds-DNA antibody levels determined by ELISA. Data are means ± SD (*N* = 7–9, *P < 0.05, ***P < 0.001, two-tailed Wilcoxon rank sum test). **H** Urinary albumin creatine level at 4 and 8 weeks, as determined by ELISA. Data are means ± SD (*N* = 6–8, *P < 0.05, two-tailed Wilcoxon rank sum test). **I** Relative expression of *IFN-α* mRNA in the spleens (left) and splenic pDCs (right) of control and IMQ mice (4 weeks), as determined by qRT-PCR. Data are means ± SD (*N* = 4–8, *P < 0.05, two-tailed Wilcoxon rank sum test). **J** miRNA expression profiling of pDCs from 4-week-old control mice and 4-week-old IMQ mice (*N* = 4 per group, fold change > 1.5). Color scales are normalized along each row. **K** Validation of downregulated miRNAs by qRT-PCR. Data are means ± SD (*N* = 8–12, **P < 0.01, ***P < 0.001, two-tailed Wilcoxon rank sum test). **L** Relative fold changes and mean expression levels of miRNAs in pDCs from 4-week-old control mice and 4-week-old IMQ mice (*N* = 3 per group), determined by small RNA-seq analysis

The imiquimod (IMQ)-induced SLE mouse model is a simple inducible animal model of SLE, in which epicutaneous treatment with the TLR7 agonist IMQ causes systemic inflammation via pDC stimulation [24]. After IMQ administration (Fig. 1A), mice developed ascites and splenomegaly at 8 weeks (Fig. 1B, C). At 4 weeks, IgG, IgM, and C3 deposition was observed in the glomeruli of IMQ mice (Fig. 1D, middle panel), whereas obvious lupus nephritis was not observed by light and electron microscopy (Fig. 1E, F, middle panel). At 8 weeks, a wire loop lesion (Fig. 1E, lower panel, black arrow), mesangial proliferation (Fig. 1E, lower panel, yellow arrow), and endocapillary hypercellularity (Fig. 1E, lower panel, arrowhead) were observed by PAS staining (Fig. 1E). Transmission electron microscopy revealed electron-dense deposits in the subepithelial (blue arrow), subendothelial (red arrowhead), and mesangial (red arrow) areas (Fig. 1F). These microscopic features were the same as those seen in human lupus nephritis. Serum anti-dsDNA antibody levels were significantly increased at 4 weeks and consistently higher in IMQ mice relative to control mice (Fig. 1G). At 8 weeks, the urine albumin creatinine ratio was significantly increased in the IMQ mice (Fig. 1H). The IFN-α mRNA levels in the spleen and splenic pDCs were elevated in IMQ mice at 4 weeks (Fig. 1I). Therefore, the SLE phenotype began to appear around 4 weeks, and organ injury was established by 8 weeks.

To examine the miRNA profile of pDCs at the onset of SLE, we purified pDCs from the spleen at 4 weeks and initially performed miRNA microarray analysis [25]. We focused on miRNAs with a fold change (FC) greater than 1.5 and selected the miRNAs for which signals were detected in all samples (Fig. 1J and Additional file 2: Table S1). We verified the downregulation of these miRNAs by qRT-PCR, except for poorly conserved miRNAs (e.g., mmu-miR-488-5p, mmu-miR-670-5p, mmu-miR-6240, mmu-miR-7662-3p). The expression of miR-128-3p and miR-148a-3p was consistently decreased in pDCs from IMQ mice relative to control mice (Fig. 1K and Additional file 1: Fig. S1). We further confirmed these results by performing a small RNA-seq analysis [25] (Fig. 1L). Importantly, small RNA-seq revealed reductions of miR-128-3p and miR-148a-3p with the high and intermediate expression levels, respectively, suggesting substantial impacts of the deregulation of two miRNAs on target regulation.

### Combinatorial inhibition of inflammatory response by miR-128 and miR-148a

**Fig. 2:**
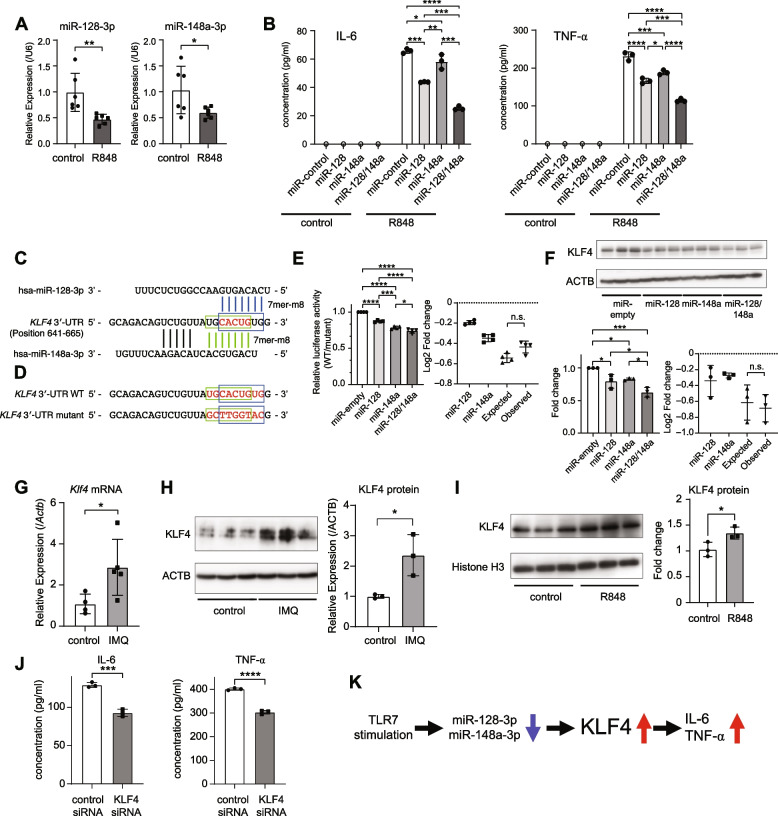
Negative regulation of inflammatory responses by miR-128-3p and miR-148a-3p through combinatorial suppression of KLF4. **A** Relative expression of miR-128-3p and miR-148a-3p in CAL-1 cells treated with R848 for 72 hours (*N* = 6, **P < 0.01, two-tailed Wilcoxon rank sum test). **B** Effect of miRNA overexpression on R848-induced cytokine production in CAL-1 cells. At 24 h after transfection with miRNAs (Additional file 1: Fig. S3A), CAL-1 cells were stimulated by R848 for 24 h and subjected to ELISA to determine the concentrations of IL-6 (left) and TNF-α (right) in the supernatant (control: *N* = 1, R848: *N* = 3, *P < 0.05, **P < 0.01, ***P < 0.001, ****P < 0.0001, one-way ANOVA and post hoc Tukey test). **C** Sequence alignments of miR-128-3p, miR-148a-3p, and their putative binding sites in the *KLF4* 3′ UTR. The seed complementary site for each miRNA is indicated by a rectangle (blue: miR-128-3p, green: miR-148a-3p). The overlap sequence is highlighted in red. **D** WT and mutant target sites of *KLF4* 3′ UTR used for luciferase reporter assay. **E** Combinatorial effects of miR-128 and miR-148a overexpression on *KLF4* 3′ UTR, as determined by luciferase reporter assay in HeLa cells. HeLa cells were transfected with 3′ UTR reporter plasmid, pRL-TK plasmid, and empty or pri-miRNA expression plasmids and subjected to luciferase assay. Relative luciferase activities for WT 3′ UTR were normalized by those for mutant 3′ UTR (left). The right panel shows the comparison of log_2_ fold change of expected repression (log2-additive) and observed repression. Data are means ± SD. Each dot represents the mean of five biological replicates in four independent experiments (*P < 0.05, ***P < 0.001, ****P < 0.0001, one-way ANOVA and post hoc Tukey test (left); n.s., not significant, two-tailed Wilcoxon rank sum test (right)). **F** Effects of miR-128 and miR-148a overexpression on KLF4 protein levels in HeLa cells. HeLa cells were transfected with pri-miRNA empty or overexpression plasmids. At 48 h after transfection (Additional file 1: Fig. S3B), western blotting analyses were performed (a representative image in the left panel). The right panel shows the comparison of log_2_ fold change of expected repression (log2-additive) and observed repression. Each dot represents the mean of a biological triplicate in three independent experiments (*P < 0.05, ***P < 0.001, one-way ANOVA and post hoc Tukey test (left); n.s., not significant, two-tailed Wilcoxon rank sum test (right)). **G** Relative expression of *Klf4* mRNA in pDCs of 4-week-old IMQ mice. Data are means ± SD (control: *N* = 4, IMQ: *N* = 5, *P < 0.05, two-tailed Wilcoxon rank sum test). **H** Western blotting analysis of KLF4 protein levels in pDCs from IMQ and control mice. Data are means ± SD (*N* = 3 per group, *P < 0.05, two-tailed Student’s *t*-test). **I** Western blotting analysis of KLF4 expression in CAL-1 cells stimulated with R848 for 72 h. Data are means ± SD (*N* = 3 per group, *P < 0.05, two-tailed Student’s *t*-test). **J** Effect of KLF4 knockdown on the inflammatory response. CAL-1 cells were transfected with control siRNA and siRNA for *KLF4*. At 24 h after transfection, cells were stimulated by R848 for 24 h and subjected to ELISA to determine the concentrations of IL-6 (left) and TNF-α (right) in the supernatant. Data are means ± SD (*N* = 3 per group, ***P < 0.001, ****P < 0.0001, two-tailed Student’s *t*-test). **K** Summary of the contribution of the miR-128/148a-KLF4 axis in pDCs to SLE pathogenesis

To investigate the direct effect of TLR stimulation on miR-128-3p and miR-148a-3p, we stimulated human leukemic pDC-like cell line CAL-1 [26] with the TLR7/8 agonist resiquimod (R848) after confirming pDC surface markers (Additional file 1: Fig. S2). The expression levels of miR-128-3p and miR-148a-3p were downregulated after 72 h of stimulation with R848 (Fig. 2A). Next, we examined the impact of miR-128-3p and miR-148a-3p on the inflammatory responses in CAL-1 cells. We introduced miR-128-3p and miR-148a-3p into CAL-1 cells by electroporation (Additional file 1: Fig. S3A) and measured R848-mediated secretion of IL-6 and TNF-α, since CAL-1 cells can secret TNF-α but not IFN-α possibly due to the tumorigenic origin [26]. Importantly, a combination of miR-128-3p and miR-148a-3p inhibited IL-6 and TNF-α production by R848 more potently than single miRNAs in CAL-1 cells (Fig. 2B). These results indicate that combinatorial downregulation of miR-128-3p and miR-148a-3p constitutes a feedback loop enhancing the TLR7-mediated inflammatory response.

### “Seed overlap” cotargeting of KLF4 by miR-128 and miR-148a

Potent suppression of inflammatory responses by miR-128-3p and miR-148a-3p (Fig. 2B) prompted us to investigate the hypothesis that two miRNAs share target(s) important for inflammation control. According to TargetScan [27], we found that the 3′ UTR of transcription factor *KLF4* has one conserved 7mer-m8 site for both miR-128-3p and miR-148a-3p (Fig. 2C). Although its role in inflammation is cell type-dependent [28, 29], KLF4 has been reported to potentiate proinflammatory signaling (NF-κB signaling) and the secretion of TNF-α and IL-6 by macrophages and synoviocytes [30–32]. Importantly, the two sites in the *KLF4* 3′ UTR extensively overlapped due to sequence similarity between the seed sequences of miR-128-3p and miR-148a-3p (Fig. 2C). To determine whether *KLF4* is a target of miR-128-3p and miR-148a-3p, we performed a 3′ UTR reporter assay using wild-type (WT) and mutant 3′ UTRs and western blotting analysis in HeLa cells (Fig. 2D–F and Additional file 1: Fig. S3B). For this purpose, we used HeLa cells because the endogenous expression of miR-128-3p and miR-148-3p was low in these cells [33]. Overexpression of two miRNAs suppressed *KLF4* 3′ UTR activity and the KLF4 protein level in HeLa cells more potently than overexpression of single miRNAs suppressed (Fig. 2D–F). We used log additivity to calculate expected fold changes using measurements with single miRNAs and compared these values to those observed for two miRNA overexpression to assess potential cooperativity (Fig. 2E, F, right). Comparisons in 3′ UTR reporter assay and western blotting analysis suggested near additive but not cooperative effects of two miRNAs (Fig. 2E, F). While observed repression tended to be lower than expected repression in the 3′ UTR reporter assay, competitive effects were not evident (Fig. 2E).

We further analyzed the role of KLF4 in the SLE model and CAL-1 cells. In pDCs from IMQ mice, we observed an increase in the *Klf4* mRNA and KLF4 protein levels (Fig. 2G, H). In addition, the KLF4 protein level was significantly increased in CAL-1 cells after 72 h of stimulation with R848 (Fig. 2I). Furthermore, knockdown of KLF4 suppressed the induction of IL-6 and TNF-α by R848 and phenocopied the effects of miR-128 and miR-148a overexpression (Fig. 2J). Therefore, miR-128 and miR-148a additively target KLF4 by extensively overlapping target sites, and the endogenous miR-128/-148a-KLF4 axis is operative in the IMQ model and CAL-1 cells (Fig. 2K).

### Increased susceptibility of “seed overlap” cotargets to downregulation by two miRNAs

**Fig. 3:**
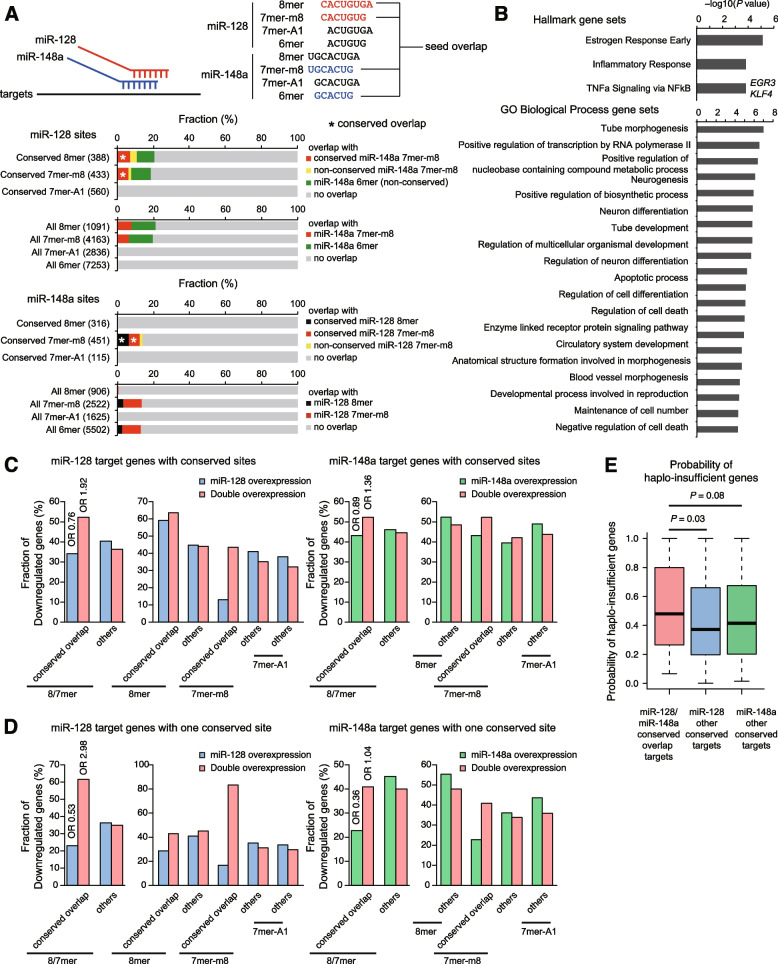
Comprehensive analysis of target responses to the combination of miR-128-3p and miR-148a-3p overexpression. **A** Conserved target sites (top) and all target sites (bottom) of miR-128-3p and miR-148a-3p with respect to “conserved overlap.” Numbers in parentheses indicate the numbers of target sites. **B** Gene set enrichment analysis of “conserved overlap” target genes performed using the hallmark gene sets and Gene Ontology biological process gene sets in the MsigDB database. *P* values were calculated using a hypergeometric test. **C**, **D** Proportions of significantly downregulated genes in distinct target groups. Results for all target genes with conserved sites (C) and target genes with one conserved site (D), and no non-conserved sites are shown. For “conserved overlap” and other 8/7mer targets (8mer and 7mer targets), odds ratios (ORs) are also shown. **E** Probability of haplo-insufficient “conserved overlap” genes and other targets. *P* values were calculated by the one-tailed Wilcoxon rank sum test

We next performed RNA-seq analysis to investigate transcriptome-wide responses of “seed overlap” cotarget sites such as *KLF4* among other sites, upon overexpression of miR-128 and miR-148a [25]. RNA-seq analysis of HeLa cells transfected with a single miRNA or two miRNAs yielded reproducible results and revealed the combined effects of the two miRNAs (Additional file 1: Fig. S4A). As expected, hierarchal suppression of 8mer > 7mer (7mer-m8 and 7mer-A1) > 6mer target genes was observed for each miRNA (Additional file 1: Fig. S4B, C). Double transfection repressed each target to a similar degree (Additional file 1: Fig. S4B, C). A comprehensive analysis of the target sites of miR-128 and miR-148a showed that 5–15% of conserved 8mer and 7mer-m8 sites of miR-128-3p and conserved 7mer-m8 sites of miR-148a-3p overlap (“conserved overlap”) (Fig. 3A). Thereafter, we focused on “conserved overlap” target sites. Target genes with such “conserved overlap” target sites were enriched in pathways related to the inflammatory response, TNF-α signaling via NF-κB, and transcriptional regulators, consistent with *KLF4* regulation by two miRNAs (Fig. 3B).

In contrast to closely spaced two target sites, “seed overlap” cotarget sites cannot be bound by two miRNAs simultaneously. Consistent with this, fold changes of genes with “seed overlap” cotarget sites did not markedly differ between the overexpression of single miRNAs and two miRNAs (data not shown). However, the proportion of significantly downregulated genes among “conserved overlap” targets increased at the expense of a slight decrease in downregulated genes among other targets, for both miRNA-128 and miR-148a target genes (Fig. 3C). In addition, a comparison of 8mer and 7mer-m8 targets of miR-128 revealed that 7mer-m8 targets were preferentially downregulated by double overexpression (Fig. 3C). These trends became more apparent when analyzing genes with only one conserved site and no non-conserved sites (Fig. 3D). Given the reported association between miRNA target site conservation and haplo-insufficient genes [34], we also evaluated the probability of haplo-insufficient genes according to a previous report [35]. Based on a comparison of haplo-insufficient and haplo-sufficient genes, the probability of haplo-insufficiency for about 17,000 genes was inferred based on conservation between human and macaque (dN/dS), promoter conservation, embryonic expression, and network proximity to known haplo-insufficient genes [35]. “Conserved overlap” target genes tended to show a higher probability of haplo-insufficiency than other target genes (Fig. 3E). *KLF4* had a high probability of being haplo-insufficient (0.84). Although “conserved overlap” sites cannot be bound by two miRNAs simultaneously, our results collectively suggest that “conserved overlap” sites increase susceptibility to overall downregulation by two distinct miRNAs through additive recruitment of two miRNAs to these sites.

### Deep conservation of “seed overlap” cotarget sites of miR-128 and miR-148a

**Fig. 4:**
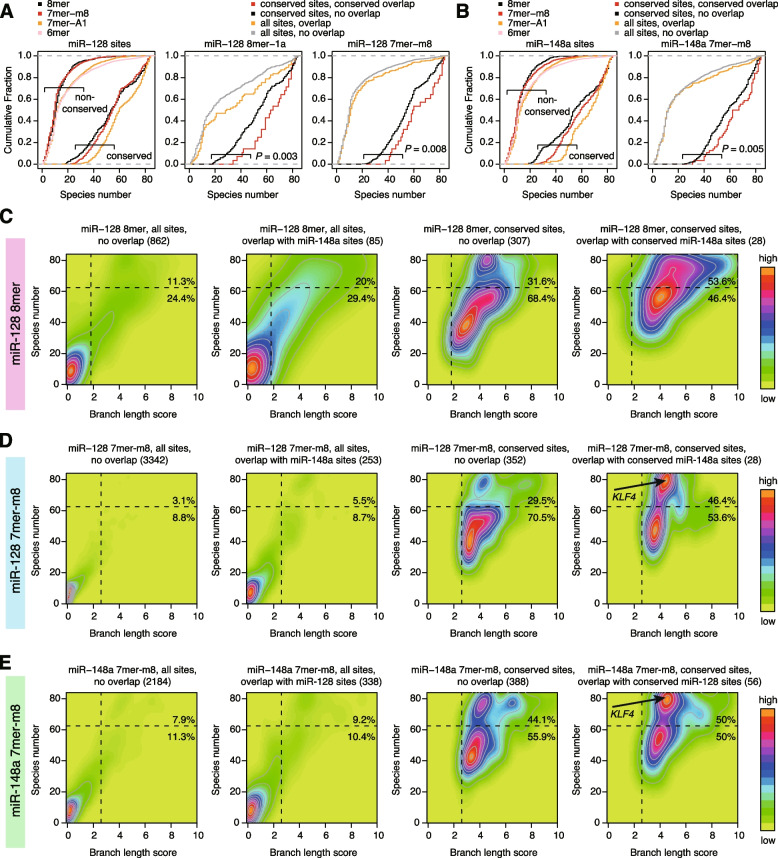
Conservation trends of miR-128 and miR-148a target sites with “conserved overlap.” **A**, **B** Cumulative distribution of the number of species in which the indicated site types of miR-128-3p (**A**) and miR-148a-3p (**B**) are conserved across 84 species. Conserved and non-conserved sites are defined by TargetScan. Target sites are classified according to Fig. 3A. *P* values were calculated by the one-tailed Wilcoxon rank sum test. **C**–**E** Density and contour plots showing the distribution of BLS values and the number of species in which sites are conserved. The results for all sites with no overlap, all sites with overlap, conserved sites with no overlap, and conserved sites with “conserved overlap” of miR-128-3p 8mer (**C**), miR-128-3p 7mer-m8 (**D**), and miR-148a-3p 7mer-m8 (**E**) are shown. Vertical and horizontal dashed lines indicate BLS cutoffs and the species number threshold (*n* = 62), respectively. Numbers in parentheses are the numbers of target sites

We further investigated the unique features of “conserved overlap” sites. During these attempts, we found that the “conserved overlap” site of *KLF4* 3′ UTR is deeply conserved across most species between human and *Coelacanth* (Additional file 1: Fig. S5). We thus investigated the conservation patterns of target sites of miR-128-3p and miR-148a-3p in humans. For miRNA target prediction, the Branch Length Score (BLS), a measure of the conservation of functional motifs across many related species, was initially introduced in the *Drosophila* study [36, 37] and subsequently incorporated into TargetScan [38]. TargetScan uses different BLS cutoffs for 8mer, 7mer-m8, and 7mer-A1 sites and distinguishes “highly conserved” (conserved) and “poorly conserved” (non-conserved) sites. Because the BLS values in TargetScan are calculated based on the phylogenetic tree of each 3′ UTR, target sites with different diversities of 3′ UTRs can show the same BLS values [38]. Therefore, we evaluated the BLS values and number of species in which the sites were conserved across 84 vertebrate species, from humans to *Coelacanth*. Fish species were excluded from 100-way multiz alignments due to poor alignment quality [27]. Because the site types (8mer, 7mer-m8, and 7mer-A1) showed slightly different numbers of conserved species (Fig. 4A, B, left), we analyzed them separately. Importantly, we found that all of miR-128 8mer, miR-128 7mer-m8, and miR-148a 7mer-m8 sites with “conserved overlap” were deeply conserved relative to other sites (Fig. 4A, B). Comparison of BLS values and numbers of conserved species confirmed these findings and further revealed that conserved sites are divisible into two classes (conserved in < and ≥ about 60 species) and that the number of conserved species is more useful for distinguishing these patterns than BLS values (Fig. 4C–E).

### Unique conservation trend of highly conserved sites of broadly conserved miRNAs

**Fig. 5:**
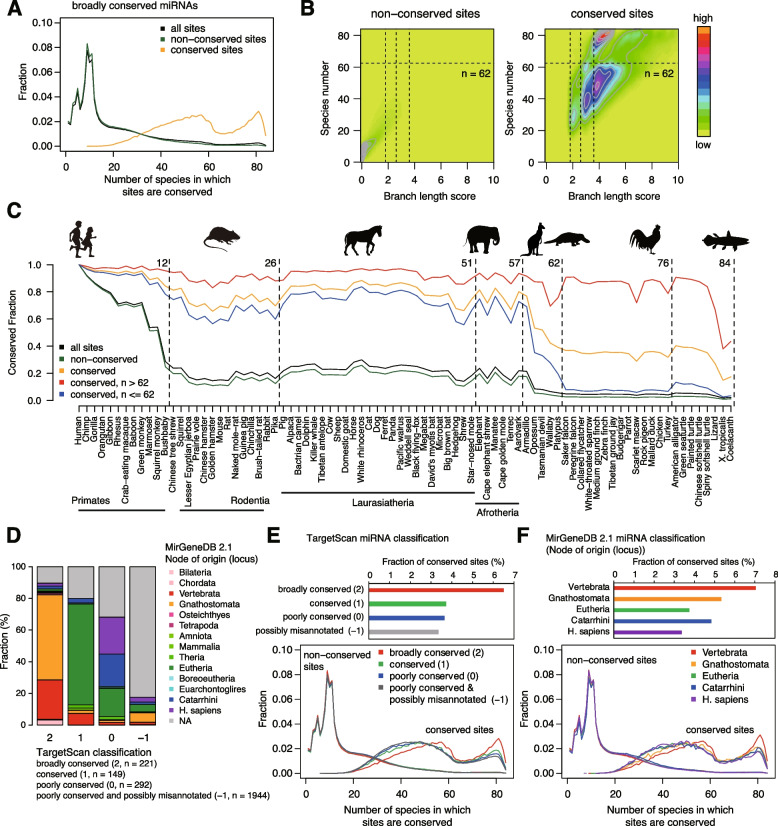
Evolutionary trends of target sites of broadly conserved miRNAs. **A** Fraction of the number of species in which “non-conserved” and conserved sites of all broadly conserved miRNAs are conserved. **B** Density and contour plots showing the distribution of BLS values and the number of species in which sites are conserved, as in **A**. Vertical and horizontal dashed lines indicate BLC cutoffs (1.8 for 8mer, 2.8 for 7mer-m8, and 3.6 for 7mer-A1) and the species number threshold (*n* = 62), respectively. **C** Conservation patterns of non-conserved sites, conserved sites, and two classes of conserved sites across 84 vertebrate species (according to the species number threshold (*n* = 62)). **D** Comparison of TargetScan miRNA classification and MirGeneDB miRNA classification (evolutionary nodes of origin (locus)). **E**, **F** Fraction of the number of species in which “non-conserved” and conserved sites for each miRNA group in TargetScan classification (**E**) and MirGeneDB classification (**F**, five major groups: Vertebrata, Gnathostomata, Eutheria, Catarrhini, and *H. sapiens*) are conserved. The top panels show the fraction of highly conserved sites for each miRNA group

Based on our findings, we expanded our analysis to all miRNA sites, especially focusing on broadly conserved miRNAs, which are conserved across most vertebrates, usually to zebrafish [27]. We first examined whether the evolutionary patterns of conserved sites are recapitulated for broadly conserved miRNAs (Fig. 4). Comparison of non-conserved and conserved sites of all broadly conserved miRNAs revealed that “non-conserved” sites are typically conserved in fewer than 20 species and that conserved sites are classified into two classes (conserved in < and ≥ about 60 species, as stated above) (Fig. 5A, B). The “summits” of the contour plot aligned vertically, indicating that BLS values cannot distinguish these two classes (Fig. 5B). Setting the threshold to 62 species, which separates *Platypus* and birds, we found that “conserved” target sites conserved in less than 62 species (65% of conserved sites) and at least 62 species (35% of conserved sites) were typically conserved among eutherian mammals and between humans and *Coelacanth*, respectively (Fig. 5C). The analysis revealed two major classes of miRNA sites.

We also compared two miRNA classification systems, TargetScan and MirGeneDB [39]. In TargetScan, miRNAs are classified as broadly conserved (group 2), conserved (group 1), poorly conserved but confidently annotated (group 0), and poorly conserved and possibly misannotated as a miRNA (group − 1). For human miRNAs, TargetScan classification and evolutionary nodes of origin in MirGeneDB are largely consistent: broadly conserved miRNAs (group 2) in TargetScan mainly correspond to the Vertebrata and Gnathostomata groups, conserved miRNAs (group 1) in TargetScan mainly correspond to the Eutheria group, and poorly conserved but confidently annotated miRNAs (group 0) in TargetScan correspond to the Eutheria, Catarrhini, and *H. sapiens* groups (Fig. 5D). We further analyzed the conservation trends of target sites for major miRNA groups. For major miRNA groups based on TargetScan and MirGeneDB, highly conserved sites are largely divided into two classes (conserved in < and ≥ 62 species) (Fig. 5E, F). Importantly, the proportion of “conserved” target sites conserved in 62 species or more is higher for broadly conserved miRNAs or Vertebrata and Gnathostomata miRNA groups than other groups, suggesting coevolution of broadly conserved miRNA genes and their targets (Fig. 5E, F). On the other hand, the conservation trends of two miRNA target site classes (conserved in < and ≥ 62 species) themselves do not largely differ among the miRNA groups (Additional file 1: Fig. S6). Taken together, these analyses revealed two major classes of miRNA sites.

### Extensive seed overlap is a pervasive feature of broadly conserved miRNAs

**Fig. 6:**
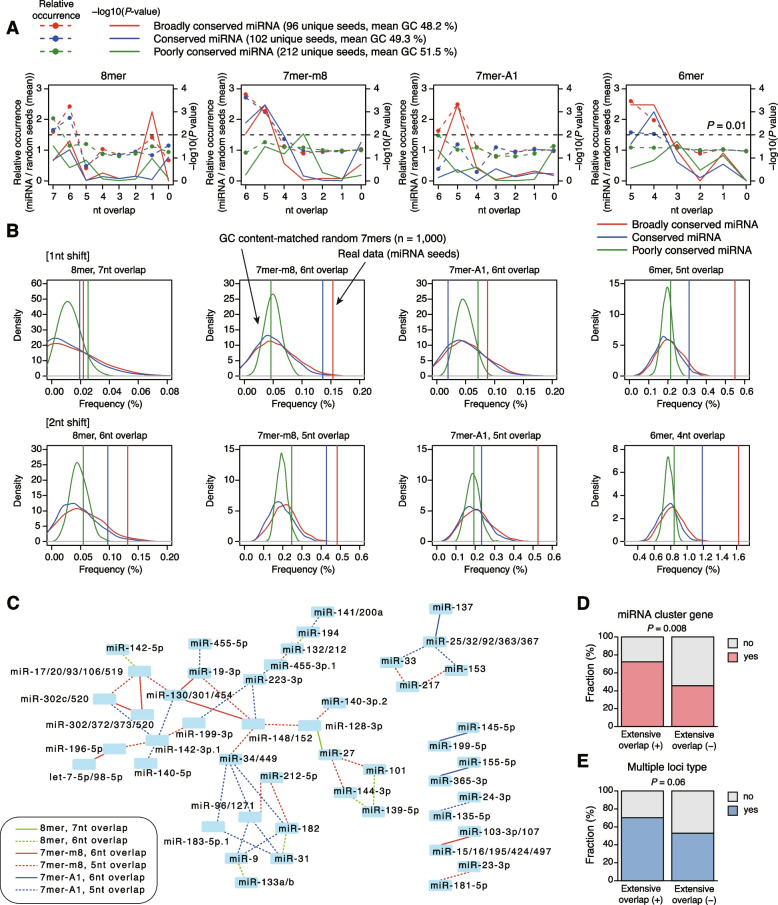
Extensive seed overlap is prevalent among broadly conserved miRNAs. **A** Summary showing relative occurrence (dashed lines) and statistics (-log10(*P* value), solid lines) of maximum overlap for each seed type (8mer, 7mer-m8, 7mer-A1, and 6mer) of all pairs of broadly conserved miRNAs, conserved miRNAs, and poorly conserved miRNAs. **B** Frequency of extensive seed overlap in real data (vertical lines) and GC content-matched random seed sequences. The results of 1000 randomizations are shown as density distributions. **C** Relationships of 50 pairs of broadly conserved miRNAs with extensive seed overlap (7/6nt overlap for 8mer and 6/5nt overlap for 7mer-m8 and 7mer-A1). **D**, **E** Associations of “extensive overlap-positive” miRNAs with miRNA cluster genes (**D**) and distribution at multiple genomic loci (**E**). *P* values were calculated by one-tailed Fisher’s exact test

We next examined whether extensive seed overlap of miRNA pairs was a pervasive feature among broadly conserved miRNAs. We analyzed three groups of miRNAs: broadly conserved miRNAs; conserved miRNAs, which are conserved across most mammals; and poorly conserved miRNAs (all other miRNAs). We compared the frequency of maximum overlap between complementary sequences of pairs of miRNAs and randomly generated seed sequences matched for G+C content. We performed 1000 randomizations and evaluated the deviation from a random distribution. The analysis revealed that 7mer-m8, 7mer-A1, and 6mer seeds of broadly conserved miRNAs tended to have a higher frequency of extensive seed overlap, i.e., 5/6 or 4/5 nt overlap for 7mer and 6mer, respectively, than expected (Fig. 6A, B and Additional file 1: Fig. S7). Although 7mer-m8 and 6mer seeds of conserved miRNAs also showed similar trends, overall, such trends were not so apparent for conserved and poorly conserved miRNAs. Figure 6C shows the relationships among 50 miRNA pairs with extensive seed overlap. miRNA genes with related sequences are frequently organized in polycistronic clusters and/or distributed at multiple genomic loci. We hypothesized that extensive seed overlap may be associated with clustering and/or multiple loci distribution of miRNA genes, to enable fine-tuning of target genes by modulating miRNA “dosage.” As expected, miRNA genes with extensive seed overlap tended to have a higher probability of being miRNA cluster genes and distributed at multiple loci (Fig. 6D, E). Consistent with this, both miR-128 and miR-148a are encoded at multiple genomic loci (miR-128-1/2 and miR-148a/b).

### Atlas of “seed overlap” miRNA cotargeting among broadly conserved miRNAs

**Fig. 7:**
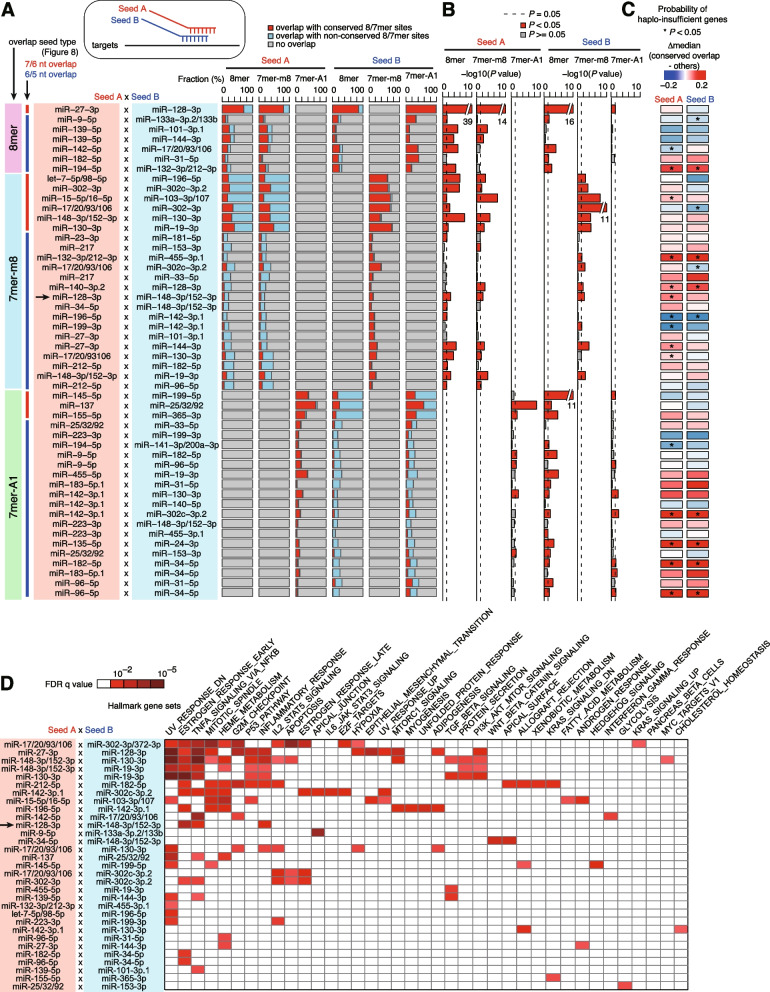
The landscape of “seed overlap” miRNA cotargeting among broadly conserved miRNAs. **A** Proportion of conserved target sites of each miRNA in 50 miRNA pairs with extensive seed overlap with respect to “conserved overlap,” as shown in Fig. 3A. Seed type and degree of overlap, shown on the left, correspond to Fig. 6A–C. A horizontal arrow indicates a pair of miR-128 and miR-148a. **B** Results of one-tailed Wilcoxon rank sum test of the conservation of target sites with “conserved overlap” relative to other target sites. Analyses were performed as in Fig. 4A, B. **C** Results of the one-tailed Wilcoxon rank sum test of the skewed probability of haplo-insufficient genes between “conserved overlap” targets and other targets. Analyses were performed as in Fig. 3E. **D** Gene set enrichment analysis of “conserved overlap” target genes performed using the hallmark gene sets in the MSigDB database. The results of the clustering analysis are shown. A horizontal arrow indicates a pair of miR-128 and miR-148a. *P* values were calculated using a hypergeometric test

To reveal the general features of the target sites of miRNA pairs with extensive seed overlap, we performed the analyses shown in Figs. 3 and 4 for 50 pairs of broadly conserved miRNAs (Fig. 7), namely analysis of the fraction of “seed overlap” cotarget sites (Fig. 7A, Additional file 2: Table S2), evolutionary trends (Fig. 7B, Fig. 3B and Additional file 2: Table S2), the probability of haplo-insufficient genes (Fig. 7C, Additional file 1: Fig. S8A), and gene set enrichment (Fig. 7D, Additional file 2: Table S3). In total, 50 pairs showed varying degrees of overlap (Fig. 7A, Additional file 2: Table S2). Importantly, as well as the miR-128 and miR-148a pair, about half of the 50 pairs showed deep conservation of “conserved overlap” target sites (Figs. 7B and Fig. 3A and Additional file 2: Table S2). The probability of haplo-insufficient genes was high for many of the 50 pairs (Fig. 7C, Additional file 1: Fig. S8B). Gene set analysis suggested that these targets may be associated with various pathways, including the inflammatory response, cell cycle, apoptosis, p53 response, and hypoxia (Fig. 7D, Additional file 2: Table S3). These results suggest that, from the standpoint of both miRNA genes and their targets, “seed overlap” miRNA cotargeting is prevalent in broadly conserved miRNAs.

### Relationships among conservation trends of target sites, miRNA cotargeting, and haplo-insufficiency of target genes

**Fig. 8:**
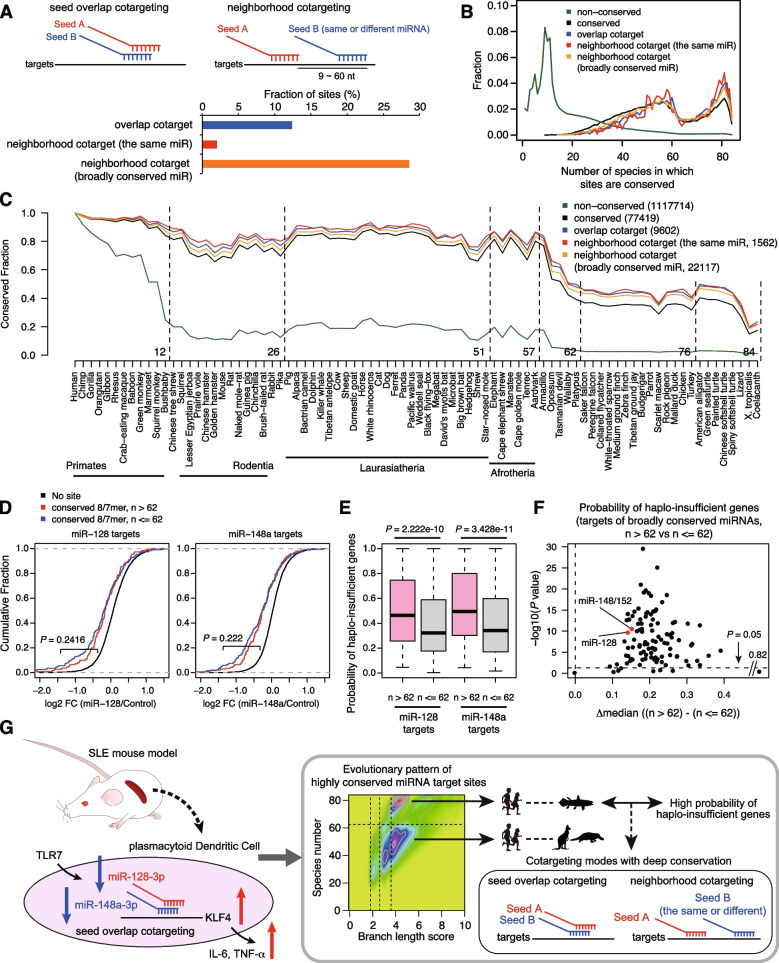
Associations of miRNA conservation classes with miRNA cotargeting, RNA regulation, and haplo-insufficiency of target genes. **A** Proportions of target sites with “seed overlap” cotargeting and “neighborhood” cotargeting among the same and different miRNAs, for all conserved target sites of broadly conserved miRNAs. **B** Fraction of the number of species in which the indicated target sites of all broadly conserved miRNAs are conserved. **C** Conservation patterns of the indicated target sites across 84 vertebrate species. **D** Cumulative distributions of fold changes (FCs) of genes with conserved 8/7mer sites of miR-128-3p (left) and miR-148a-3p (right), according to the species number threshold of 62. *P* values were calculated by the one-tailed Wilcoxon rank sum test. Numbers in parentheses are the numbers of target sites. **E** Probability of haplo-insufficient target genes with conserved 8/7mer sites of miR-128-3p (left) and miR-148a-3p (right), according to the species number threshold of 62. *P* values were calculated by the one-tailed Wilcoxon rank sum test. **F** Summary of differences and statistics of the probability of haplo-insufficient target genes with conserved 8/7mer sites, according to the species number threshold of 62. The results of all broadly conserved miRNAs are shown. *P* values were calculated by the one-tailed Wilcoxon rank sum test. **G** Summary of the present study

We finally examined how the two classes of target sites (Fig. 5) correlate with multiple modes of miRNA cotargeting, RNA repression, and target gene features. We extracted the target sites showing “seed overlap” cotargeting and “neighborhood” miRNA cotargeting of the same miRNAs or distinct miRNAs (Fig. 8A) and analyzed the evolutionary trends (Fig. 8B, C and Additional file 1: Fig. S9). Both target sites with “seed overlap” cotargeting and “neighborhood” miRNA cotargeting showed deeper conservation (Fig. 8B). Increased conservation was consistently observed for both “seed overlap” cotargeting and “neighborhood” miRNA cotargeting, from humans to *Coelacanth* (Fig. 8C). This suggests that both cotargeting sites are deeply conserved beyond eutherian mammals.

We further analyzed our RNA-seq data in terms of the downregulation of two classes of miR-128 and miR-148a targets; there was no obvious difference between the two classes (Fig. 8D). By contrast, both deeply conserved classes of miR-128 and miR-148a had a high probability of haplo-insufficient genes (Fig. 8E). When extended to all broadly conserved miRNAs, strikingly, the deeply conserved classes of most miRNAs had a high probability of haplo-insufficiency (Fig. 8F). Taken together, the deeply conserved class has a stronger association with both “seed overlap” and “neighborhood” miRNA cotargeting and a higher probability of haplo-insufficient genes. Therefore, even if target efficiency does not markedly differ between two target site classes, an association with multiple cotargeting modes and the dose sensitivity of target genes may lead to different functional consequences.

## Discussion

In the present study, we have demonstrated that two miRNAs, miR-128 and miR-148a, are coordinately downregulated in pDCs in the IMQ-induced SLE mouse model and that downregulation of miR-128-3p and miR-148a-3p constitutes a feedback loop that enhances the TLR7-mediated inflammatory response by additively targeting KLF4 (Fig. 8G). Consistent with our findings, a previous study in patients with SLE, SLE and antiphospholipid syndrome (APS), and primary APS has described the lower expression of miR-128-3p in patients with a high IFN signature relative to patients with a low IFN signature and healthy controls [23]. Our recent preliminary analysis of blood samples from SLE patients also suggested downregulation of miR-128-3p (data not shown). While alterations in miR-148a expression levels and the functional importance appear to be cell type-dependent in SLE and other autoimmune diseases [40–42], our results are consistent with a prior report that miR-148 is a negative regulator of the innate immune response in DCs [43]. Therefore, miR-128 and miR-148a in pDCs in combination may have the potential as a diagnostic or prognostic biomarker of inflammatory activity in SLE. Given that modulation of certain immune-related miRNAs, including miR-146a and miR-155, can ameliorate inflammatory conditions such as sepsis-induced organ injury and lupus alveolar hemorrhage in mice [44, 45], the therapeutic potential of miR-128 and miR-148a for SLE warrants further investigation.

The miR-128 and miR-148a pair may also have important roles in other diseases. A meta-analysis of genome-wide association studies (GWAS) revealed that multiple miRNAs in physical proximity to single nucleotide polymorphisms (SNPs) are associated with abnormal circulating lipids [46, 47]. These miRNAs include miR-128-1, miR-148a, miR-130b, and miR-301b, which synergistically regulate cholesterol and triglyceride homeostasis. Importantly, our analysis suggests that these miRNAs engage in crosstalk via extensive seed overlap (between miR-148 and miR-130/301, and between miR-128 and miR-148; Fig. 6C). Multifaceted interactions between “seed overlap” miRNAs and lipid-associated SNPs may be associated with transcriptional mechanisms controlling these miRNAs in a modular manner. Although the mechanisms underlying the coordinated downregulation of miR-128 and miR-148a in SLE are unclear, miR-148a and miR-128-2 are proposed to be transcriptional targets of Nrf2, a key regulator of cellular response to oxidized phospholipids and antioxidant response, in endothelial cells and heart [48, 49]. In line with these reports, Nrf2 has been reported to suppress lupus nephritis through inhibition of oxidative injury and the NF-κB-mediated inflammatory response in mice [50–52]. Thus, alteration in lipid metabolism and Nrf2 activities may be associated with the downregulation of the two miRNAs.

Numerous studies have described the dysregulation of multiple miRNAs in various diseases, and some have investigated their combined effects [53, 54]. However, the significance of miRNA cotargeting is largely unclear in these settings. In contrast to “seed overlap” cotargeting, the mechanism of “neighborhood” cotargeting has been well-studied [3–6]. Mechanistically, synergistic effects of closely spaced sites can be explained by multivalent biding of TNRC6 proteins (also known as GW182 proteins) to multiple miRNA-Argonaute (Ago) complexes [55]. This mode of cotargeting appears to be deeply conserved; the GW182 protein in *Nematostella* is a mediator of miRNA-mediated silencing [56]. Consistent with this, we detected deep conservation of “neighborhood” cotargeting across 84 vertebrate species (Fig. 8B, C).

The coexpression of multiple related miRNAs from polycistronic miRNA genes is known to mediate the coordinated regulation of target genes in the related pathways [57]. From a mechanistic standpoint, overlap or excessively close spacing between two sites exerts a steric hindrance effect precluding the binding of a second miRNA-Ago complex [55]. Consistent with this, in the previous reports, very closely spaced pairs of target sites of identical or distinct miRNAs showed less efficient or equivalent repression relative to single sites [3–5]. By contrast, in a previous study on the miR-15/16 families, which bind to CTG repeats and frequently exhibit extensively overlapping sites for the same miRNA, mRNA repression reportedly increased as the number of pairs of extensively overlapping sites increased [6]. A similar scenario has been proposed for isomiR-mediated cotargeting [58]. Our experiments demonstrated that KLF4 is additively downregulated by miR-128 and miR-148a. Our RNA-seq analyses further demonstrated the responses of “seed overlap” cotarget sites. Although the fold changes of genes with “seed overlap” cotarget sites did not markedly differ according to whether one or two miRNAs were overexpressed, “seed overlap” cotarget sites increased susceptibility to overall downregulation by two miRNAs. Therefore, “seed overlap” cotarget sites may be preferred to single sites, especially in the presence of two miRNAs. These effects would depend on whether the overlap cotarget sites were targeted by the same miRNA or different miRNAs: the latter scenario would more efficiently increase susceptibility to target repression by expanding the miRNA pools that can be recruited to the sites, while such sites cannot be bound by two miRNAs simultaneously and the target efficiency for each miRNA can be attenuated due to competition between two miRNAs. In addition, such additive effects would be attenuated when the relative concentrations of two miRNAs become higher than those of target RNAs and two miRNAs become more competitive. Further analyses with miRNA dose titration would shed lights on the details of kinetics of “seed overlap” miRNA cotargeting. Furthermore, our analysis also suggests that “intermediate” sites (7mer sites) are more susceptible to this type of regulation than “strong” sites (8mer sites). This suggests that “intermediate” sites gain more benefits from increased dosage effects of two miRNAs than “strong” sites. Collectively, these findings suggest that “seed overlap” cotarget sites can increase target efficiency although less effective than two closely spaced sites.

Although “seed overlap” cotargeting is less effective than “neighborhood” cotargeting, integrated bioinformatics revealed that extensive seed overlap of miRNA genes and deep conservation of “seed overlap” cotarget sites are prevalent among broadly conserved miRNA genes and their targets. Deep conservation of both “seed overlap” cotargeting and “neighborhood” cotargeting (Fig. 8) may suggest that multiple evolutionary mechanisms stabilize miRNA networks. For typical miRNA target sites, evolutionary maintenance of the A at position 1, which enhances target repression through enhanced interactions with Ago2, and enriched local AU contents in the region flanking the seed sequences contribute to increased site efficiency [5, 59]. In terms of usage of these beneficial sequence contexts, “seed overlap” sites are frequently constrained due to the requirement of seed sequence overlap. For example, in the case of miR-128 and miR-148a pair, 8mer target sites of miR-148a cannot overlap with target sites of miR-128, and one side of the franking sequences of miR-148a is restricted by the seed sequences of miR-128. Therefore, deep conservation of “seed overlap” sites may be associated with distinct evolutionary trajectories independent of simple enhancement of target repression, as others suggested that miRNA target site conservation is complicated [34]. Given that “seed overlap” cotargeting is not inherently cooperative, it is possible that “seed overlap” cotargeting mainly contributes to the robustness of target repression through compensation among multiple miRNAs. Combinatorial loss of related miRNAs could preferentially deregulate “seed overlap” cotargets. The association of “seed overlap” cotargeting with miRNA cluster genes, distribution at multiple genomic loci, and a high probability of haplo-insufficient genes suggests that fine-tuning the “dosage” of both miRNAs and target mRNAs is important for this type of regulation and may have been positively selected during evolution [60].

Another important finding of this study was the systematic identification of two major conservation classes of highly conserved sites of broadly conserved miRNAs and other miRNAs (Fig. 8G). This is consistent with a previous report suggesting two large “evolutional dips,” from fish to warm-blooded vertebrates and from birds to eutherian mammals [61]. Using both the number of conserved species and BLS values as inputs may improve the performance of current miRNA target prediction programs, including TargetScan. While current TargetScan is based on improved 3′ UTR annotations [27], further improvements in 3′ UTR annotations and integration of the evolutionary history of miRNA genes (represented in MirGeneDB) may update our findings [39]. In our analysis, target repression did not markedly differ between the two classes, which may be explained by equivalent biochemical affinities. However, the deeply conserved target sites of most conserved miRNAs exhibit a high probability of haplo-insufficient genes. This provides insight into the biological functions of miRNAs beyond the simple interpretation of RNA repression. It is an important open question how these distinct classes of miRNA target sites choreograph the biological functions of miRNAs. These findings may also improve our understanding of newly evolved sites and of newly evolved or mutant miRNA genes [62, 63]. Taken together, this study sheds light on complex aspects of miRNA cotargeting and miRNA target site conservation.

## Conclusions

In this study, we report the pathogenic impacts of “seed overlap” miRNA cotargeting in SLE. Integrative analyses further demonstrated that “seed overlap” miRNA cotargeting is a prevalent feature of both deeply conserved miRNAs and their target sites, and importantly uncovered two major conservation classes of target sites, those conserved among eutherian mammals and between humans and *Coelacanth*. The latter has a stronger association with both “seed overlap” and “neighborhood” miRNA cotargeting and implicates higher dosage sensitivity. These findings highlight the importance of perturbed miRNA cotargeting in human pathology and unique evolutionary aspects of miRNA cotargeting and miRNA target site conservation.

## Methods

### Mouse model

FVB/NJcl female mice were purchased from Clea (Shizuoka, Japan). We employed the imiquimod (IMQ)-induced SLE model [24]. Briefly, the skin on the right ear of mice aged 7–9 weeks was treated topically, three times weekly, with 1.25 mg of 5% IMQ cream (Mochida Pharmaceutical, Tokyo, Japan). Vaseline was used as the control. Mice were sacrificed at 4 or 8 weeks. All mouse experiments were performed according to the protocols approved by the Institutional Animal Care and Use Committee of Nagoya University (protocol number 30459).

### Histology

Kidney tissues were fixed in formalin, embedded in paraffin, cut into 4 μm sections, and stained with Periodic acid-Schiff reagent. For immunofluorescence, the sections of kidney tissues were embedded in OCT compound, frozen, cut into 4 μm sections, and stained with anti-IgG (1:100) (115-095-166, Jackson Immuno Research, West Grove, PA), anti-IgM (1:100) (1020-02, SouthernBiotech, Birmingham, AL), and anti-C3 (1:3200) (55500, MP Biomedicals, Irvine, CA) antibodies. For transmission electron microscopy, kidney tissues were double-fixed in 2.5% glutaraldehyde and 2% osmium tetroxide. The tissues were embedded in Epon 812. Ultrathin sections were generated using an ULTRACUT S (Leica, Germany) and double-stained with uranyl acetate and lead. Images were obtained using a transmission electron microscope (JEM-1400 Ex; JEOL, Tokyo, Japan) at 60 kV.

### Purification of plasmacytoid DCs from mice

The spleens were dissociated into single-cell suspensions using the Spleen Dissociation Kit (Miltenyi Biotec, NRW, Germany) according to the manufacturer’s instructions. From this suspension, we purified pDCs using the Plasmacytoid Dendritic Cell Isolation Kit (Miltenyi Biotec) according to the manufacturer’s instructions. We confirmed that the purified cells were pDCs—i.e., were B220-positive, PDCA1-positive, and CD11c-intermediate—by fluorescence-activated cell sorting [64].

### miRNA microarray

Total RNA was extracted from isolated pDCs using the miRNeasy Micro Kit (Qiagen, Hilden, Germany) according to the manufacturer’s instructions. We determined the RNA integrity numbers (RINs) using a bioanalyzer (Agilent, Santa Clara, CA); samples with RINs greater than 8.0 were used for miRNA Array analysis (TORAY 3D-gene CHIP, Kanagawa, Japan). miRNA array data were analyzed by GeneSpring (Tommy-digital, Tokyo, Japan) [25]. We searched for potential target genes of selected miRNAs using TargetScan (v7.2) [27].

### Small RNA sequencing

Total RNA was extracted from isolated pDCs using the miRNeasy Micro Kit (Qiagen, Hilden, Germany) according to the manufacturer’s instructions. Small RNA libraries were generated using the NEB Next Multiplex Small RNA Library Prep Set for Illumina (New England Biolabs, Ipswich, MA) and analyzed by Illumina NovaSeq 6000 [25]. Sequence analysis was performed according to the previous reports [9, 62, 65, 66]. Obtained sequences were processed for adapter removal and size exclusion of sequences < 15 nt with Cutadapt. Filtered reads were mapped to the mm10 genome assembly with bowtie 1.0.1, allowing two mismatches, and further quantitated using miRbase v21.

### qRT-PCR analysis

Total RNA was extracted from cell and tissue samples using TRIzol Reagent (Thermo Fisher Scientific, Waltham, MA). For the detection of miRNAs, total RNA was reverse-transcribed using the TaqMan MicroRNA Reverse Transcription Kit (Thermo Fisher Scientific) and analyzed using the TaqMan MicroRNA Assays (Thermo Fisher Scientific). For the detection of mRNAs, total RNA was reverse-transcribed using the QuantiTect Reverse Transcription Kit (Qiagen). Targets were amplified by real-time PCR with a Step One Plus Real-Time PCR System (Thermo Fisher Scientific) and IDT Master Mix (Integrated DNA Technologies) or Fast Advanced Master Mix (Thermo Fisher Scientific). All samples were assayed in duplicate. Quantitative evaluation of target expression was performed by the ΔΔCT method. miRNA and mRNA expression levels were normalized to those of U6 snRNA and β-actin (Actb), respectively. Primer sequences are listed in Additional file 2: Table S4.

### Cell culture and transfection

Human leukemic pDC-like cells (CAL-1) were described in a previous report [26]. CAL-1 cells were cultured in RPMI1680 (Sigma Aldrich, St. Louis, MO) supplemented with 10% heat-inactivated fetal bovine serum at 37 °C in 5% CO_2_. For western blotting and enzyme-linked immunosorbent assay (ELISA), CAL-1 cells were plated in a six-well plate (0.5 or 1.0 × 10^5^/ml). CAL-1 cells were stimulated with 1 μg/ml of the TLR7 agonist R848 (InvivoGen, San Diego, CA). The control siRNA (Silencer™ Select Negative Control No. 1 siRNA, #4390843) and siRNA for *KLF4* (s17794) were purchased from Thermo Fisher Scientific. We confirmed reproducible results using multiple siRNAs for *KLF4*. miRNA-control (mirVana™ miRNA Mimic Negative Control #1, #4464058) and miRNA mimics of miR-128 (MC11746) and miR-148a (MC10263) were purchased from Thermo Fisher Scientific. For siRNA and miRNA transfection, CAL-1 cells were suspended in an appropriate amount of electroporation buffer. Next, 0.4 ml of cell suspension containing 1.25 × 10^7^ cells/ml and 100 nM oligonucleotide was transferred into a 4-mm electroporation cuvette. The Gene Pulser Xcell System (Bio-Rad, Berkeley, CA) was used for single-cuvette electroporation. The electroporation conditions were exponential mode, 300 V, and 100 μF.

HeLa cells were purchased from the American Type Culture Collection and maintained in DMEM (Sigma Aldrich) supplemented with 10% heat-inactivated fetal bovine serum at 37 °C in 5% CO_2_. For transfection experiments, HeLa cells were plated in a six-well plate (1.5 × 10^5^ cells per well). After overnight culture, transfection was performed using Lipofectamine 2000 (Thermo Fisher Scientific) according to the manufacturer’s instructions. According to miRmine [33], a database of miRNA expression profiles in various cell lines, we focused on cell lines with low expression of miR-128-3p and miR-148a-3p, including HeLa-S3; confirmed their low expression in HeLa cells by qRT-PCR analysis; and used them for subsequent experiments.

### Flow cytometric analysis

Mice pDCs and CAL-1 cells were analyzed using a FACS Canto II instrument (BD Biosciences, San Jose, CA) in conjunction with the FlowJo software (TreeStar, Ashland, OR). The monoclonal antibodies were as follows: anti-human CD11c (301617), anti-human CD304 (354503), anti-human CD123 (306011), anti-mouse/human CD45R/B220 (103225), anti-mouse CD317 (BST2, PDCA-1) (127104), and anti-mouse CD11c (117310) (all from BioLegend, San Diego, CA).

### Plasmids

Pri-miRNA expression vectors were designed according to a previous report [67]. Briefly, pri-miRNA expression vectors were generated by cloning short fragments of pri-miRNAs containing pre-miRNA and a flanking sequence on both sides of the pre-miRNA, within the NheI and XhoI sites of the multi-cloning site of pcDNA3.1(+) (Thermo Fisher Scientific). We used pcDNA3.1(+) as the negative control. For 3′ UTR reporter assay, the full-length 3′ UTR of human *KLF4* mRNA (NCBI Nucleotide accession number NM_001314052.2) was cloned into the pMIR-REPORT™ Luciferase vector (Thermo Fisher Scientific) using NEBuilder HiFi Assembly (New England Biolabs). Mutagenesis of *KLF4* 3′ UTR was performed by inverse PCR. Primer sequences are listed in Additional file 2: Table S5.

### Enzyme-linked immunosorbent assay

Levels of cytokines (IL-6, TNF-α) were measured using a Quantikine ELISA Kit (R&D Systems, Minneapolis, MN). The anti-dsDNA antibody level was measured using the Levis anti-dsDNA Mouse ELISA Kit (Fujifilm, Tokyo, Japan). An Albumin ELISA Kit (Albuwell M; Ethos Biosciences, Newtown Square, PA) and Creatinine Assay Kit (Cayman Chemical Company, Ann Arbor, MI) were used to measure the urinary albumin:creatinine ratio. All samples were assayed in duplicate.

### Western blotting

Western blotting analysis was performed as described previously [44]. Briefly, cellular proteins were separated by 7% or 15% SDS-PAGE and transferred onto PVDF membranes (Whatman, Florham Park, NJ). The membranes were blocked with 5% (wt/vol) dry fat-free milk in TBS-T buffer (0.1% Tween) for 60 min at room temperature. Next, the membranes were incubated with rabbit anti-human KLF4 (4038, Cell Signal Technology, Danvers, MA), rabbit anti-histoneH3 (4499, Cell Signal Technology), or rabbit anti-β-actin (ACTB) antibodies (4967, Cell Signal Technology) in TBS-T buffer (5% BSA, 0.1% Tween) at 4 °C overnight. After washing with TBS-T buffer (0.1% Tween), the membranes were incubated with the goat anti-rabbit IgG HRP-linked secondary antibody (7074, Cell Signal Technology) at room temperature for 60 min. Proteins were visualized using an enhanced chemiluminescence (ECL) detection system (Amersham Pharmacia, Piscataway, NJ). Unprocessed images of western blots are shown in Additional file 1: Fig. S10.

### Luciferase assay

HeLa cells were seeded in a 24-well plate (4.0 × 10^4^ cells per well). After overnight culture, HeLa cells were transfected with 25 ng of 3′ UTR reporter plasmid, 25 ng of pRL-TK plasmid (Promega, Madison, WI), and 350 ng of various combinations of pcDNA3.1(+) empty or pri-miRNA expression plasmids using Lipofectamine 2000, according to the manufacturer’s instructions. At 48 h after transfection, cells were lysed, and luciferase activities were measured using SpectraMax® iD5 Multimode Microplate Reader (Molecular Devices, San Jose, CA) and Dual-Luciferase Reporter Assay System (Promega). The ratio of Firefly luciferase activity to Renilla luciferase activity was calculated for each well.

### RNA-seq analysis

For RNA-seq, HeLa cells were plated in a six-well plate (1.0 × 10^5^ per well). After overnight culture, HeLa cells were transfected with miR-control 20 nM (control), miR-128-3p 10 nM + miR-control 10 nM (miR-128), miR-148a-3p 10 nM + miR-control 10 nM (miR-148a), or miR-128-3p 10 nM + miR-148a-3p 10 nM (miR-128/148a) by Lipofectamine RNAiMAX (Thermo Fisher Scientific). At 48 h after transfection, total RNA was extracted using the miRNeasy Micro Kit. We checked the RINs of RNA using a bioanalyzer; samples with RINs greater than 8.0 were used for RNA-seq. RNA-seq experiments involved three biological replicates. Libraries for RNA-seq were prepared using the SMART-Seq v4 Ultra Low Input RNA Kit for Sequencing (Clontech, Mountain View, CA) and Nextera XT DNA Library Prep Kit (Illumina, San Diego, CA), and were subjected to paired-end sequencing using the NovaSeq 6000 instrument (Illumina) [25]. The sequencing reads were aligned to the reference genome (hg38) using STAR (v2.5.3) [68]. Reads on each RefSeq gene were counted with HTSeq (v0.6.0) in intersection-strict mode [69], and the edgeR package in R was used to identify differentially expressed genes with a false discovery rate (FDR) threshold of 0.05 or 0.1 [70]. After filtering out the genes with a maximum count per million (CPM) across all samples of less than 1, the trimmed mean of the *M*-value normalization (TMM) method and generalized linear models were used to analyze the gene expression data. Principal component analysis and *K*-means clustering were performed on the top 1000 most variable genes. Differentially expressed genes were grouped into six clusters by *K*-means clustering. Downregulated genes were defined as genes with fold changes (FCs) < 0 and FDR < 0.05. Gene set overlap analysis was performed using the hallmark gene sets, miRNA target gene sets, and Gene Ontology (GO) biological process (BP) gene sets in the MsigDB database (https://www.gsea-msigdb.org/gsea/msigdb/) and the hypergeometric test.

### miRNA information and seed overlap analysis

Information on human miRNAs was downloaded from the TargetScan Human database (v7.2 [27]; http://www.targetscan.org/vert_72/) in May 2021. Classification of miRNAs (broadly conserved (2), conserved (1), poorly conserved but confidently annotated (0), and poorly conserved and possibly misannotated as a miRNA (− 1)) was done according to TargetScan information. We classified poorly conserved but confidently annotated miRNAs (group 0) as poorly conserved miRNAs. To reduce overlap between isomiRs, miRNAs with no MiRBase Accession ID were excluded. Information on evolutionary nodes of origin (locus information) for miRNA genes was from MirGeneDB database (v2.1 [39]; https://mirgenedb.org/). We combined target sites of group 2, 1, and 0 miRNAs in TargetScan and grouped them according to MirGeneDB information. For seed overlap analysis (Fig. 6), we evaluated the maximum overlap of complementary sequences of each site type for all pairs of miRNA groups and the same number of random seed sequences that matched the G+C content distribution. We performed 1000 randomizations and assessed the statistical significance. Information on miRNA cluster genes and the distribution of miRNA genes at multiple genomic loci was obtained from miRbase [71].

### Analysis and classification of target sites

Target site predictions in the present study were based on the latest version of TargetScan Human (v7.2). Based on the Branch Length Score (BLS) cutoffs for each site type, conserved sites and “conserved overlap,” i.e., overlap between two conserved sites, were defined. In addition to BLS values, we analyzed another metric of conservation, i.e., the number of species in which target sites are conserved. To do this, we used multiple species alignments in TargetScan and determined the number of species for each site across 84 species [27]. The probability of haplo-insufficient genes was described in a previous report [35].

### Statistical analysis

Statistical tests were performed using GraphPad Prism (9.0.2, GraphPad; GraphPad Software Inc., San Diego, CA) and R (4.0.1; R Development Core Team). In Fig. 2B, E (left), F (left) and Additional file 1: Fig. S3A, B, statistical analysis was performed using one-way analysis of variance (ANOVA) and post hoc Tukey test. In Figs. 1G–I, K; Fig. 2 A, E (right), F (right), G; 3E; 4A, B; 7B, C; and 8D–F and Additional file 1: Fig. S4B-C and Fig. S8A-B, statistical analysis was performed using the one-tailed or two-tailed Wilcoxon rank sum test. In Fig. 2H–J, statistical analysis was performed using the two-tailed Student’s *t*-test. In Fig. 3B and Fig. 7D, statistical analysis was performed using a hypergeometric test. In Fig. 6D, E, *P* values were calculated by the one-tailed Fisher’s exact test. In all bar graphs, data are means ± SD. In all box plots, center lines show the medians; box limits indicate the twenty-fifth and seventy-fifth percentiles; whiskers extend to 1.5× the interquartile range. We independently repeated the molecular biology experiments at least twice, and all attempts to reproduce the results were successful.

## Supplementary Information

**Additional file 1: Fig. S1.** Additional results of qRT-PCR analysis. Additional validation of downregulated miRNAs by qRT-PCR. Data are means ± SD (N = 8-12). **Fig. S2.** Characterization of the human pDC-like cell line CAL-1. **(A-C)** FACS analysis of the cell-surface markers CD11c (A), CD304 (B), and CD123 (C) in CAL-1 cells. Isotype controls are shown in blue. **Fig. S3.** Overexpression of miR-128 and miR-148a in CAL-1 and HeLa cells. **(A)** Overexpression of miR-128-3p and miR-148a-3p in CAL-1 cells. Transfection with miR-control, miR-128-3p and miR-control, miR-148a-3p and miR-control, or miR-128-3p and miR-148a-3p, followed by qRT-PCR analysis (N = 3 per group, ***P < 0.001, ****P < 0.0001, one-way ANOVA and post hoc Tukey test). **(B)** Overexpression of miR-128-3p and miR-148a-3p in HeLa cells. HeLa cells were transfected with pri-miRNA empty or overexpression plasmids. At 48 hours after transfection, qRT-PCR analyses were performed (N = 3 per group, **P < 0.01, ***P < 0.001, one-way ANOVA and post hoc Tukey test). **Fig. S4.** Transcriptomic effects of miR-128 and miR-148a overexpression in HeLa cells. **(A)** Principal component analysis (left) and K-means clustering analysis (right) of the 1,000 most variable genes in RNA-seq datasets. The bottom panel shows enrichment of miR-128-3p and mIR-148a-3p target genes in each DEG group, classified by K-means clustering analysis. **(B, C)** Cumulative distributions of fold changes (FCs) of genes with single 8mer, 7mer-m8, 7mer-A1, and 6mer sites of miR-128-3p (B) and miR-148a-3p (C) upon single or double miRNA transfection. *P* values for downregulation (vs. genes with no sites) were calculated by one-tailed Wilcoxon rank sum test. **Fig. S5.** Deep conservation of *KLF4* seed overlap cotarget sites between human and *Coelacanth*. TargetScan sequence alignments around the seed overlap cotarget site within *KLF4* 3′ UTRs are shown. **Fig. S6.** Conservation trends of miRNA target sites based on TargetScan and MirGeneDB classification. **(A)** Density and contour plots showing the distribution of BLS values and number of species in which the sites are conserved. Results for target sites of major miRNA groups in TargetScan (top) and MirGeneDB (bottom) classification are shown. **(B)** Conservation patterns of non-conserved sites, conserved sites, two classes of conserved sites for major miRNA groups in TargetScan (top) and MirGeneDB (bottom) classification across 84 vertebrate species (according to the species number threshold (n = 62)). **Fig. S7.** Additional seed overlap analysis of miRNA genes. **(A)** Summary showing relative occurrence (dashed lines) and statistics (-log10(*P* value), solid lines) of the maximum overlap for each seed type (8mer, 7mer-m8, 7mer-A1, and 6mer) among all pairs of (1) broadly conserved miRNAs, (2) broadly conserved and conserved miRNAs, and (3) broadly conserved, conserved, and poorly conserved miRNAs. Note that the results were similar when different miRNA groups were combined. **(B)** Frequency of extensive seed overlap in real data (vertical lines) and GC content-matched random seed sequences. Results of 1,000 randomizations are shown as density distributions. **Fig. S8.** Analysis of evolutionary trends in “seed overlap” miRNA cotargets and probability of haplo-insufficient genes. **(A)** Summary of differences and statistics of the number of species in which the sites are conserved. Target sites with “conserved overlap” and other target sites were compared. The results for the 50 miRNA pairs shown in Figure 7 are shown by site type. *P* values were calculated by one-tailed Wilcoxon rank sum test for either direction. **(B)** Summary of differences and statistics of the probability of haplo-insufficient genes between target genes with “conserved overlap” or other target sites. Results for 50 miRNA pairs shown in Figure 7 are shown. *P* values were calculated by one-tailed Wilcoxon rank sum test for either direction. **Fig. S9.** Additional seed overlap analysis of miRNA genes. Density and contour plots showing the distribution of BLS values and number of species in which the sites are conserved. Results for sites with “seed overlap” cotargeting (left), “neighborhood” cotargeting for the same miRNA (middle), and “neighborhood” cotargeting for all broadly conserved miRNAs (right) are shown. Vertical and horizontal dashed lines indicate BLC cutoffs (1.8 for 8mer, 2.8 for 7mer-m8, and 3.6 for 7mer-A1) and the species number threshold (n = 62). The trends do not markedly differ between groups. **Fig. S10.** Images of the full-size original blots. Uncropped images for Fig. 2 are shown.

**Additional file 2: Table S1.** The list of up-regulated and down-regulated miRNAs in pDCs from the IMQ mouse model (miRNA microarray analysis). **Table S2.** Summary of the number of seed overlap target sites for 50 miRNA pairs and evolutionary trends, as shown in Figure 7A and B. **Table S3.** Summary of the gene set analysis performed using hallmark gene sets (Figure 7D). **Table S4.** Primer information for RT-PCR. **Table S5.** Primer information used for construction of pri-miRNA vectors and KLF4 reporter vector.

## Data Availability

miRNA microarray, small RNA-seq, and RNA-seq datasets generated in this study are available in the Gene Expression Omnibus under accession number GSE184338 [25]. Unprocessed images of western blots are shown in Additional file 1: Fig. S10.

## Abbreviations

IC: Immune complex
IFN: Interferon
IMQ: Imiquimod
KLF4: Kruppel-like factor 4
miRNA: MicroRNA
pDC: Plasmacytoid dendritic cell
SLE: Systemic lupus erythematosus
TLR: Toll-like receptor
3′ UTR: 3′ untranslated region
