# Supplementary material for: Systematic characterization of seed overlap microRNA cotargeting associated with lupus pathogenesis
Source: BMC Biol. 2022 Nov 11;20:248. doi: 10.1186/s12915-022-01447-4 (PMC9650897; doi:10.1186/s12915-022-01447-4)
Supplement: Supplementary file 1 — Additional file 1: Fig. S1. Additional results of qRT-PCR analysis. Additional validation of downregulated miRNAs by qRT-PCR. Data are means ± SD (N = 8-12). Fig. S2. Characterization of the human pDC-like cell line CAL-1. (A-C) FACS analysis of the cell-surface markers CD11c (A), CD304 (B), and CD123 (C) in CAL-1 cells. Isotype controls are shown in blue. Fig. S3. Overexpression of miR-128 and miR-148a in CAL-1 and HeLa cells. (A) Overexpression of miR-128-3p and miR-148a-3p in CAL-1 cells. Transfection with miR-control, miR-128-3p and miR-control, miR-148a-3p and miR-control, or miR-128-3p and miR-148a-3p, followed by qRT-PCR analysis (N = 3 per group, ***P < 0.001, ****P < 0.0001, one-way ANOVA and post hoc Tukey test). (B) Overexpression of miR-128-3p and miR-148a-3p in HeLa cells. HeLa cells were transfected with pri-miRNA empty or overexpression plasmids. At 48 hours after transfection, qRT-PCR analyses were performed (N = 3 per group, **P < 0.01, ***P < 0.001, one-way ANOVA and post hoc Tukey test). Fig. S4. Transcriptomic effects of miR-128 and miR-148a overexpression in HeLa cells. (A) Principal component analysis (left) and K-means clustering analysis (right) of the 1,000 most variable genes in RNA-seq datasets. The bottom panel shows enrichment of miR-128-3p and mIR-148a-3p target genes in each DEG group, classified by K-means clustering analysis. (B, C) Cumulative distributions of fold changes (FCs) of genes with single 8mer, 7mer-m8, 7mer-A1, and 6mer sites of miR-128-3p (B) and miR-148a-3p (C) upon single or double miRNA transfection. P values for downregulation (vs. genes with no sites) were calculated by one-tailed Wilcoxon rank sum test. Fig. S5. Deep conservation of KLF4 seed overlap cotarget sites between human and Coelacanth. TargetScan sequence alignments around the seed overlap cotarget site within KLF4 3′ UTRs are shown. Fig. S6. Conservation trends of miRNA target sites based on TargetScan and MirGeneDB classification. (A) Density and contour [file 12915_2022_1447_MOESM1_ESM.pdf]

Additional file 1

Systematic characterization of seed overlap microRNA cotargeting associated with lupus pathogenesis

Hiroki Kitai, Noritoshi Kato*, Koichi Ogami, Shintaro Komatsu, Yu Watanabe, Seiko Yoshino, Eri Koshi, Shoma Tsubota, Yoshio Funahashi, Takahiro Maeda, Kazuhiro Furuhashi, Takuji Ishimoto, Tomoki Kosugi, Shoichi Maruyama, Kenji Kadomatsu, Hiroshi I. Suzuki*

*Corresponding author. Email: hisuzuki@med.nagoya-u.ac.jp (H.I.S.); n-kato@med.nagoya-u.ac.jp (N.K.)

Additional file 1

Figure S1-10

Figure S1

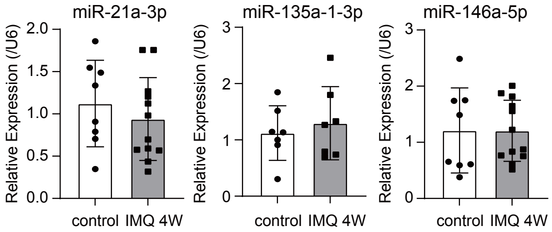

Figure S1. Additional results of qRT-PCR analysis.
Additional validation of downregulated miRNAs by qRT-PCR. Data are means ± SD (N = 8-12).

Figure S2

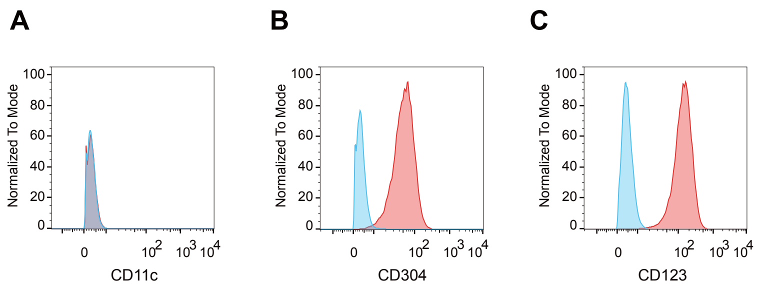

Figure S2. Characterization of the human pDC-like cell line CAL-1.

(A-C) FACS analysis of the cell-surface markers CD11c (A), CD304 (B), and CD123 (C) in CAL-1 cells. Isotype controls are shown in blue.

Figure S3

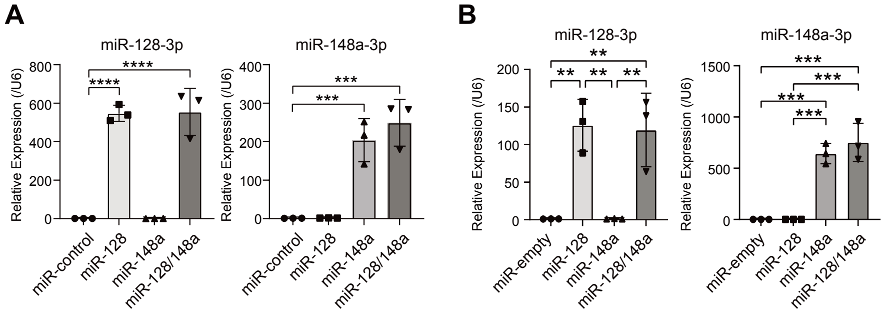

Figure S3. Overexpression of miR-128 and miR-148a in CAL-1 and HeLa cells.

(A) Overexpression of miR-128-3p and miR-148a-3p in CAL-1 cells. Transfection with miR-control, miR-128-3p and miR-control, miR-148a-3p and miR-control, or miR-128-3p and miR-148a-3p, followed by qRT-PCR analysis (N = 3 per group, ***P < 0.001, ****P < 0.0001, one-way ANOVA and post hoc Tukey test).

(B) Overexpression of miR-128-3p and miR-148a-3p in HeLa cells. HeLa cells were transfected with pri-miRNA empty or overexpression plasmids. At 48 hours after transfection, qRT-PCR analyses were performed (N = 3 per group, **P < 0.01, ***P < 0.001, one-way ANOVA and post hoc Tukey test).

Figure S4

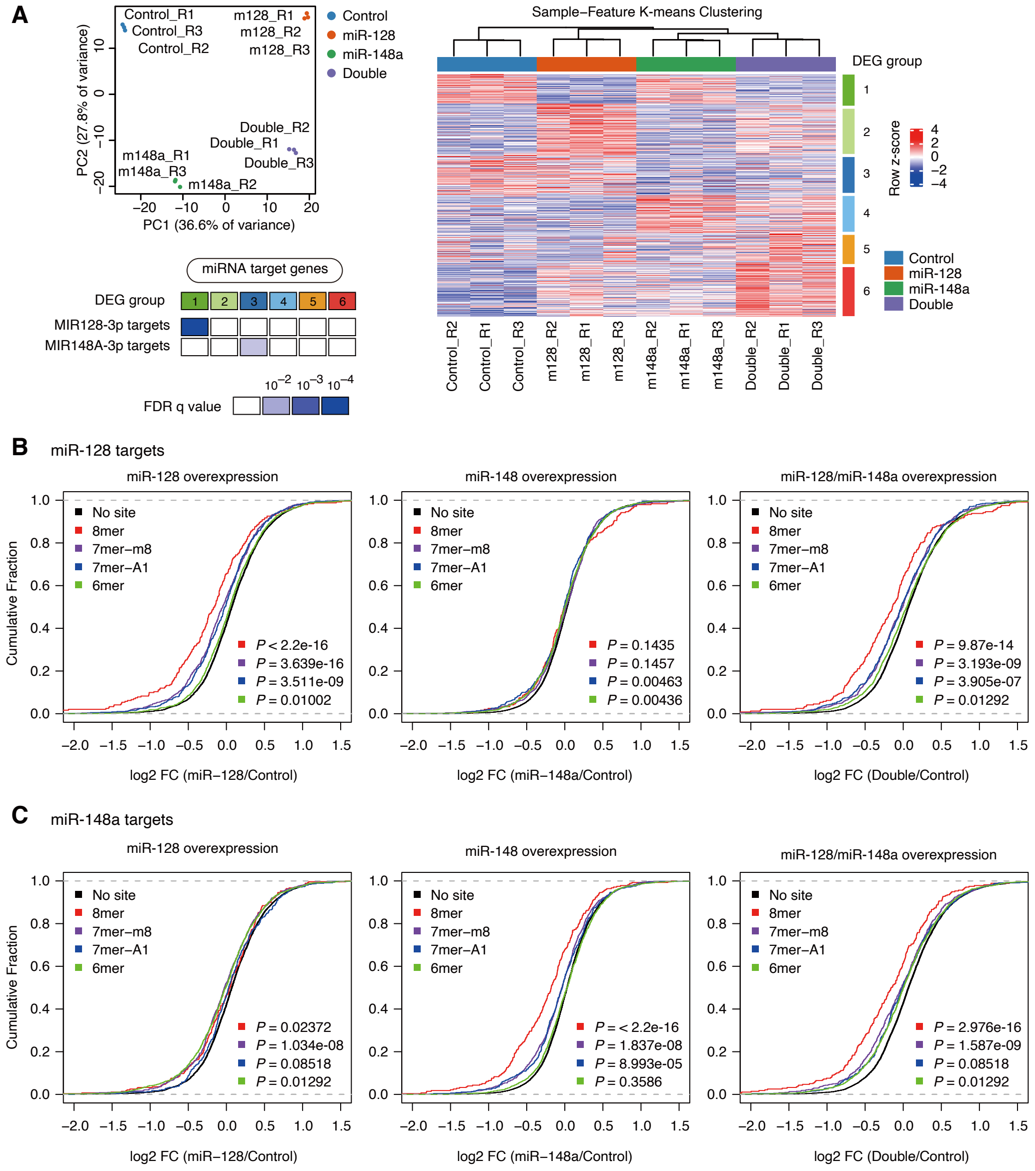

Figure S4. Transcriptomic effects of miR-128 and miR-148a overexpression in HeLa cells.

(A) Principal component analysis (left) and K-means clustering analysis (right) of the 1,000 most variable genes in RNA-seq datasets. The bottom panel shows enrichment of miR-128-3p and mIR-148a-3p target genes in each DEG group, classified by K-means clustering analysis.

(B, C) Cumulative distributions of fold changes (FCs) of genes with single 8mer, 7mer-m8, 7mer-A1, and 6mer sites of miR-128-3p (B) and miR-148a-3p (C) upon single or double miRNA transfection. P values for downregulation (vs. genes with no sites) were calculated by one-tailed Wilcoxon rank sum test.

Figure S5

miR-148a · miR-128

Species	650...........660.......670.......680
Human	U-GUU--A-**UGCACUG**UGGUUUCAGAUGUGCAAUA
Chimp	U-GUU--A-**UGCACUG**UGGUUUCAGAUGUGCAAUA
Gorilla	U-GUU--A-**UGCACUG**UGGUUUCAGAUGUGCAAUA
Orangutan	U-GUU--A-**UGCACUG**UGGUUUCAGAUGUGCAAUA
Gibbon	U-GUU--A-**UGCACUG**UGGUUUCAGAUGUGCAAUA
Rhesus	U-GUU--A-**UGCACUG**UGGUUUCAGAUGUGCAAUA
Macaque	U-GUU--A-**UGCACUG**UGGUUUCAGAUGUGCAAUA
Baboon	U-GUU--A-**UGCACUG**UGGUUUCAGAUGUGCAAUA
Green monkey	U-GUU--A-**UGCACUG**UGGUUUCAGAUGUGCAAUA
Marmoset	U-GUU--A-**UGCACUG**UGGUUUCAGAUGUGCAAUA
Squirrel monkey	U-GUU--A-**UGCACUG**UGGUUUCAGAUGUGCAAUA
Bushbaby	U-GUU--A-**UGCACUG**UGGUUUCAGAUGUGCAAUA
Tree shrew	U-GUU--A-**UGCACUG**UGGUUUCAGAUGUGCAAUA
Squirrel	U-GUU--A-**UGCACUG**UGGUUUCAGAUGUGCAAUA
Jerboa	U-GUU--A-**UGCACUG**UGGUUUCAGAUGUGCAAUA
Vole	U-GUU--A-**UGCACUG**UGGUUUCAGAUGUGCAAUA
Chinese hamster	U-GUU--A-**UGCACUG**UGGUUUCAGAUGUGCAAUA
Golden hamster	U-GUU--A-**UGCACUG**UGGUUUCAGAUGUGCAAUA
Mouse	U-GUU--A-**UGCACUG**UGGUUUCAGAUGUGCAAUA
Rat	U-GUU--A-**UGCACUG**UGGUUUCAGAUGUGCAAUA
Mole-rat	U-GUU--A-**UGCACUG**UGGUUUCAGAUGUGCAAUA
Guinea pig	U-GUU--A-**UGCACUG**UGGUUUCAGAUGUGCAAUA
Chinchilla	U-GUU--A-**UGCACUG**UGGUUUCAGAUGUGCAAUA
Brush-tailed rat	U-GUU--A-**UGCACUG**UGGUUUCAGAUGUGCAAUA
Rabbit	U-GUU--A-**UGCACUG**UGGUUUCAGAUGUGCAAUA
Pika	U-GUU--A-**UGCACUG**UGGUUUCAGAUGUGCAAUA
Pig	U-GUU--A-**UGCACUG**UGGUUUCAGAUGUGCAAUA
Alpaca	U-GUU--A-**UGCACUG**UGGUUUCAGAUGUGCAAUA
Camel	U-GUU--A-**UGCACUG**UGGUUUCAGAUGUGCAAUA
Dolphin	U-GUU--A-**UGCACUG**UGGUUUCAGAUGUGCAAUA
Whale	U-GUU--A-**UGCACUG**UGGUUUCAGAUGUGCAAUA
Antelope	U-GUU--A-**UGCACUG**UGGUUUCAAAUGUGCAAUA
Cow	U-GUU--A-**UGCACUG**UGGUUUCAGAUGUGCAAUA
Sheep	U-GUU--A-CGCAUUGUGGUUUCAGAUGUGCAAUA
Goat	U-GUU--A-**UGCACUG**UGGUUUCAGAUGUGCAAUA
Horse	U-GUU--A-**UGCACUG**UGGUUUCAGAUGUGCAAUA
Rhinoceros	U-GUU--A-**UGCACUG**UGGUUUCAGAUGUGCAAUA
Cat	U-GUU--A-**UGCACUG**UGGUUUCAGAUGUGCAAUA
Dog	U-GUU--AA**UGCACUG**UGGUUUCAGAUGUGCAAUA
Ferret	U-GUU--A-**UGCACUG**UGGUUUCAGAUGUGCAAUA
Panda	U-GUU--A-CG**CACUG**UGGUUUCAGAUGUGCAAUA
Walrus	U-GUU--A-**UGCACUG**UGGUUUCAGAUGUGCAAUA
Seal	U-GUU--A-**UGCACUG**UGGUUUCAGAUGUGCAAUA

Species	Sequence
Flying-fox	U-GUU--A-**UGCACUG**UGGUUUCAGAUGUGCAAUA
Megabat	U-GUU--A-**UGCACUG**UGGUUUCAGAUGUGCAAUA
Myotis bat	U-GUU--A-**UGCACUG**UGGUUUCAGAUGUGCAAUA
Microbat	U-GUU--A-**UGCACUG**UGGUUUCAGAUGUGCAAUA
Brown bat	U-GUU--A-**UGCACUG**UGGUUUCAGAUGUGCAAUA
Hedgehog	U-GUU--A-**UGCACUG**UGGUUUCAGAUGUGCAAUA
Shrew	U-GUU--A-**UGCACUG**UGGUUUCAAAUGUGCAAUA
Star-nosed mole	U-GUU--A-**UGCACUG**UGGUUUCAAAUGUGCAAUA
Elephant	U-GUU--A-**UGCACUG**UGGUUUCAGAUGUGCAAUA
Cape shrew	-----------------------------------
Manatee	U-GUU--A-**UGCACUG**UGGUUUCAGAUGUGCAAUA
Cape golden mole	UUGUU-UA-**UGCACUG**UGGUUUCAGAUGUGCAAUA
Tenrec	UUGUUGUA-**UGCACUG**UGGUUUCAGAUGUGCAAUA
Aardvark	UUGUUGUA-**UGCACUG**UGGUCUCAGAUGUGCAAUA
Armadillo	U-GUU-UA-AG**CACUG**--GUUUCAGAUGUGCAAUA
Opossum	U-AUA--A-**UGCACUG**UGGUUUCA-AUGUGCAAUA
Devil	U-AUA--A-**UGCACUG**UGGUUUCA-AUGUGCAAUA
Wallaby	U-AUA--A-**UGCACUG**UGGUUUCA-AUGUGCAAUA
Platypus	U-GUA--A-**UGCACUG**UGGUUUCA-AUGUGCAAUA
Saker falcon	U-GUA--A-**UGCACUG**UGGUUUC-AAUGUGCAAUA
Peregrine falcon	U-GUA--A-**UGCACUG**UGGUUUC-AAUGUGCAAUA
Flycatcher	U-GUA--A-**UGCACUG**UGGUUUC-GAUGUGCAAUA
Sparrow	U-GUA--A-**UGCACUG**UGGUUUC-GAUGUGCAAUA
Ground finch	U-GUA--A-**UGCACUG**UGGUUUC-GAUGUGCAAUA
Zebra finch	U-GUA--A-**UGCACUG**UGGUUUC-GAUGUGCAAUA
Jay	U-GUA--A-**UGCACUG**UGGUUUC-GAUGUGCAAUA
Budgerigar	U-GUA--A-**UGCACUG**UGGUUUC-GAUGUGCAAUA
Parrot	U-GUA--A-**UGCACUG**UGGUUUC-GAUGUGCAAUA
Macaw	-----------------------------------
Pigeon	U-GUA--A-**UGCACUG**UGGUUUC-GAUGUGCAAUA
Duck	U-GUA--A-**UGCACUG**UGGUUUC-GAUGUGCAAUA
Chicken	U-GUA--A-**UGCACUG**UGGUUUC-GAUGUGCAAUA
Turkey	U-GUA--A-**UGCACUG**UGGUUUC-GAUGUGCAAUA
Alligator	U-GUA--A-**UGCACUG**UGGUUUC-GAUGUGCAAUA
Seaturtle	U-GUA--A-**UGCACUG**UGGUUUC-AAUGUGCAAUA
Painted turtle	U-GUA--A-**UGCACUG**UGGUUUC-AAUGUGCAAUA
Chinese turtle	U-AUA--A-**UGCACUG**UGGUUUC-GAUGUGCAAUA
Spiny turtle	U-AUA--A-**UGCACUG**UGGUUUC-GAUGUGCAAUA
Lizard	U-GUA--A-**UGCACUG**UGGUUUC-AAUGUGCAAUA
X. tropicalis	U-GUG--A-CG**CACUG**UGGUUUC-UAUGUGCAAUA
Coelacanth	U-GUA--A-**UGCACUG**UGGCCAC-UAUGUGCAAUA
Consensus	U.GUU..A.**UGCACUG**UGGUUUCAGAUGUGCAAUA

Figure S5. Deep conservation of *KLF4* seed overlap cotarget sites between human and *Coelacanth*. TargetScan sequence alignments around the seed overlap cotarget site within *KLF4* 3′ UTRs are shown.

Figure S6

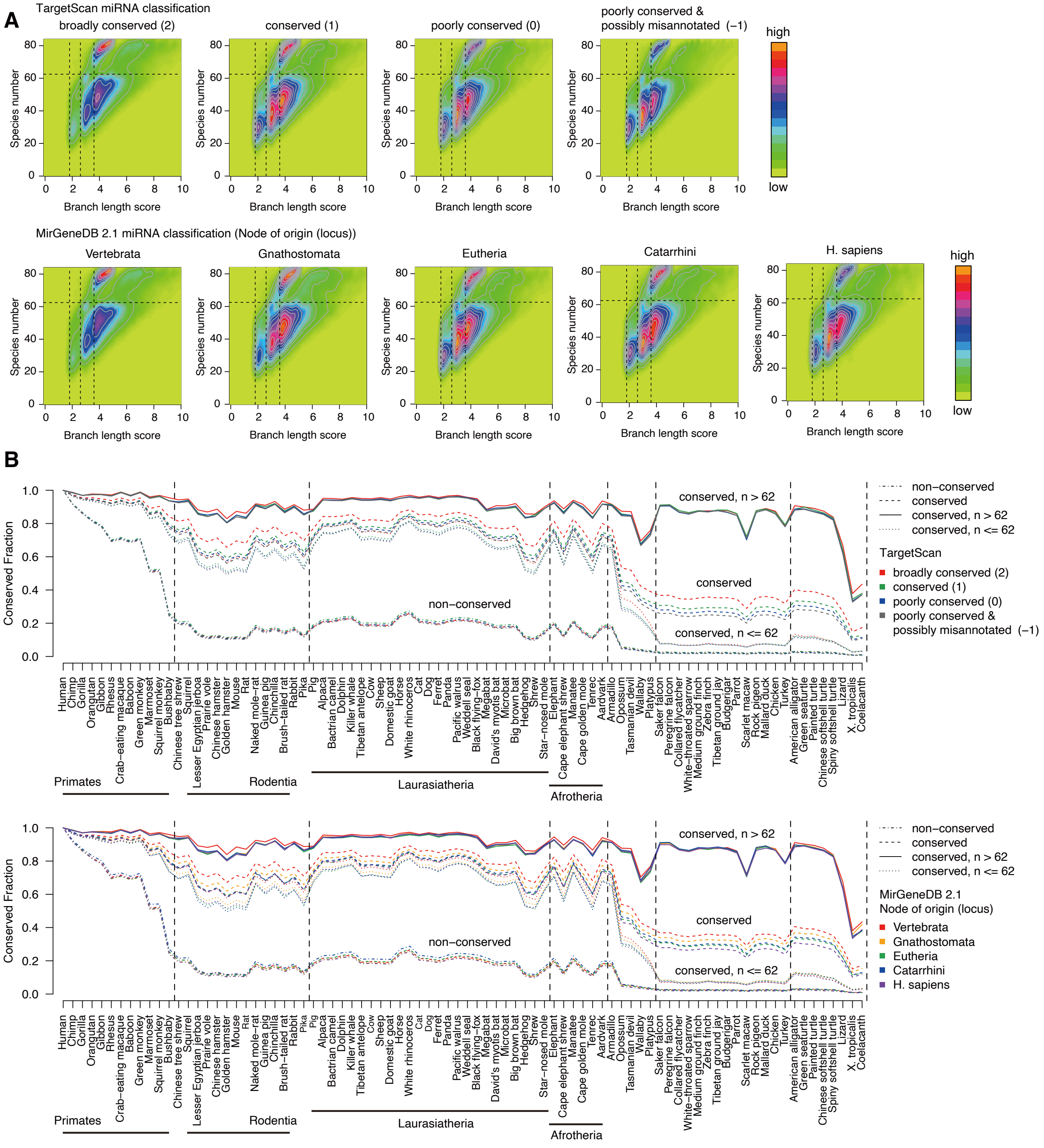

Figure S6. Conservation trends of miRNA target sites based on TargetScan and MirGeneDB classification.

(A) Density and contour plots showing the distribution of BLS values and number of species in which the sites are conserved. Results for target sites of major miRNA groups in TargetScan (top) and MirGeneDB (bottom) classification are shown.

(B) Conservation patterns of non-conserved sites, conserved sites, two classes of conserved sites for major miRNA groups in TargetScan (top) and MirGeneDB (bottom) classification across 84 vertebrate species (according to the species number threshold (n = 62)).

Figure S7

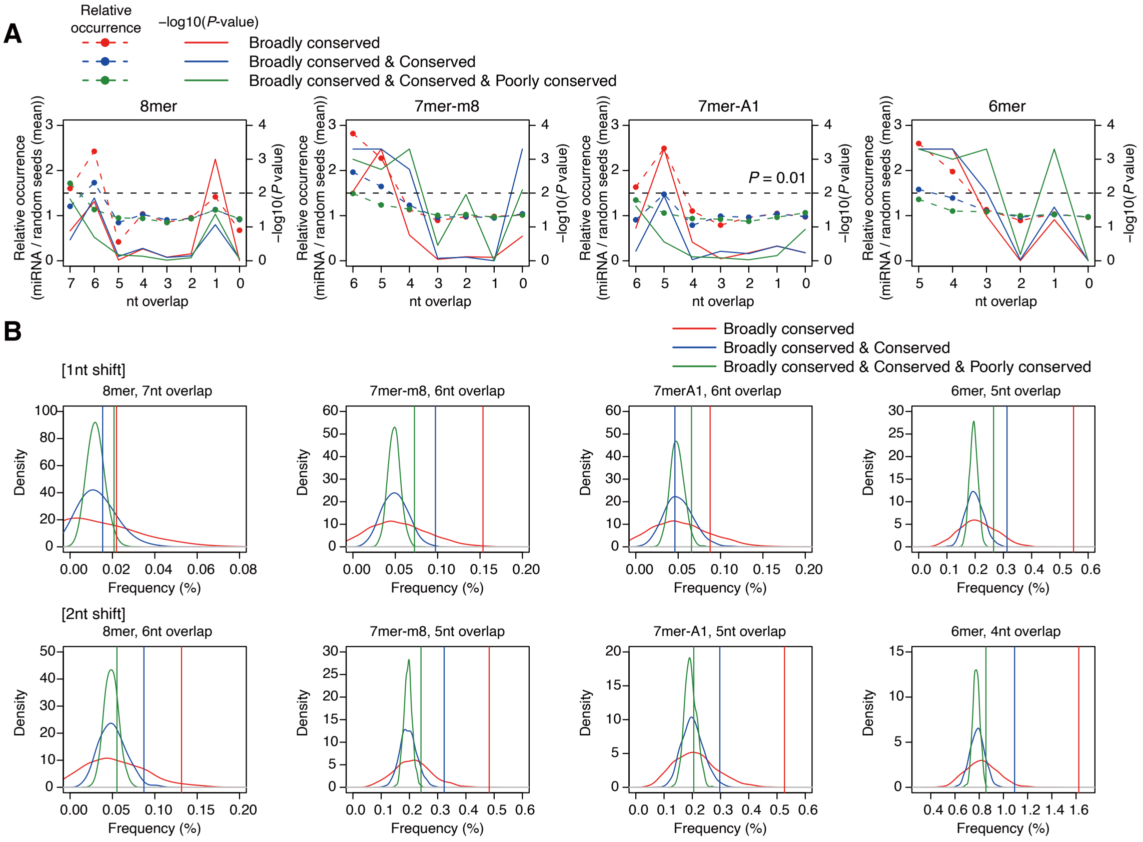

Figure S7. Additional seed overlap analysis of miRNA genes.

(A) Summary showing relative occurrence (dashed lines) and statistics (-log10(*P* value), solid lines) of the maximum overlap for each seed type (8mer, 7mer-m8, 7mer-A1, and 6mer) among all pairs of (1) broadly conserved miRNAs, (2) broadly conserved and conserved miRNAs, and (3) broadly conserved, conserved, and poorly conserved miRNAs. Note that the results were similar when different miRNA groups were combined.

(B) Frequency of extensive seed overlap in real data (vertical lines) and GC content-matched random seed sequences. Results of 1,000 randomizations are shown as density distributions.

Figure S8

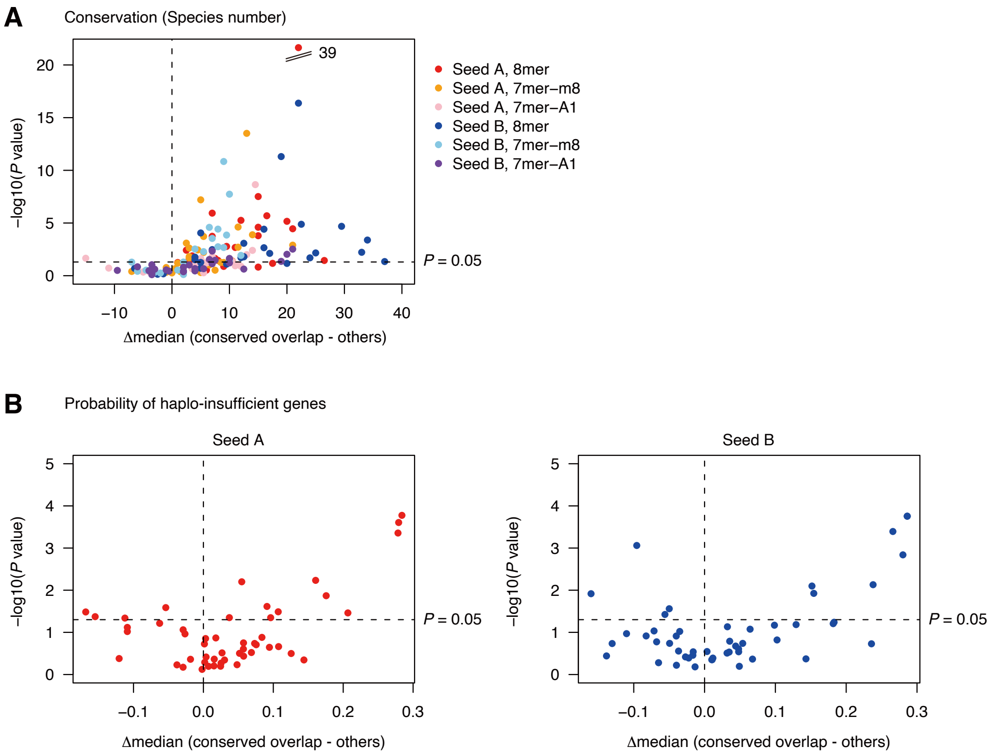

Figure S8. Analysis of evolutionary trends in "seed overlap" miRNA cotargets and probability of haplo-insufficient genes.

(A) Summary of differences and statistics of the number of species in which the sites are conserved. Target sites with "conserved overlap" and other target sites were compared. The results for the 50 miRNA pairs shown in Figure 7 are shown by site type. P values were calculated by one-tailed Wilcoxon rank sum test for either direction.

(B) Summary of differences and statistics of the probability of haplo-insufficient genes between target genes with "conserved overlap" or other target sites. Results for 50 miRNA pairs shown in Figure 7 are shown. P values were calculated by one-tailed Wilcoxon rank sum test for either direction.

Figure S9

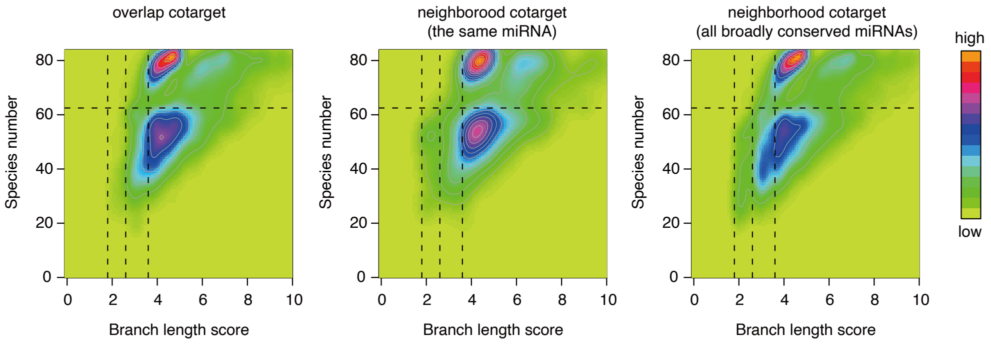

Figure S9. Additional seed overlap analysis of miRNA genes.

Density and contour plots showing the distribution of BLS values and number of species in which the sites are conserved. Results for sites with "seed overlap" cotargeting (left), "neighborhood" cotargeting for the same miRNA (middle), and "neighborhood" cotargeting for all broadly conserved miRNAs (right) are shown. Vertical and horizontal dashed lines indicate BLC cutoffs (1.8 for 8mer, 2.8 for 7mer-m8, and 3.6 for 7mer-A1) and the species number threshold (n = 62). The trends do not markedly differ between groups.

Figure S10

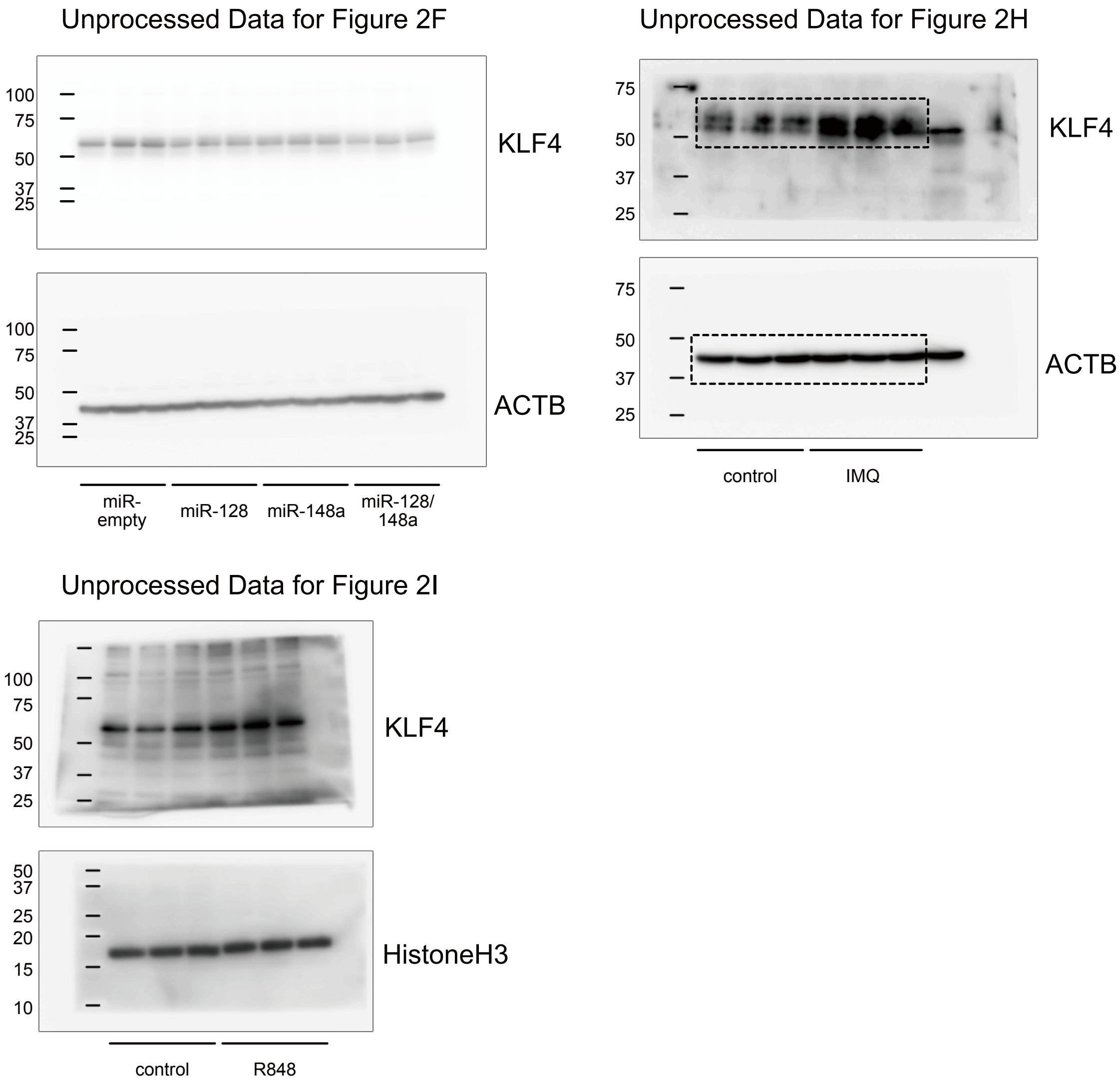

Figure S10. Images of the full-size original blots.
Uncropped images for Fig. 2 are shown.

Additional file 2

Table S1.

The list of up-regulated and down-regulated miRNAs in pDCs from the IMQ mouse model (miRNA microarray analysis).

Table S2.

Summary of the number of seed overlap target sites for 50 miRNA pairs and evolutionary trends, as shown in Figures 7A and 7B.

Table S3.

Summary of the gene set analysis performed using hallmark gene sets (Figure 7D).

Table S4.

Primer information for RT-PCR.

Table S5.

Primer information used for construction of pri-miRNA vectors and KLF4 reporter vector.
